# Polylactic-Containing Hyperbranched Polymers through the CuAAC Polymerization of Aromatic AB_2_ Monomers

**DOI:** 10.3390/ijms24087620

**Published:** 2023-04-21

**Authors:** Aurora Pacini, Andrea Nitti, Marcello Vitale, Dario Pasini

**Affiliations:** 1 Department of Chemistry, INSTM Research Unit, University of Pavia, Viale Taramelli 10, 27100 Pavia, Italy; 2 IVM Chemicals s.r.l., Viale della Stazione 3, 27020 Parona, Italy

**Keywords:** hyperbranched polymers, Cu(I)-catalyzed alkyne–azide cycloaddition polymerization, aromatic AB_2_ monomers

## Abstract

We report on the synthesis and characterization of a novel class of hyperbranched polymers, in which a copper(I)-catalyzed alkyne azide cycloaddition (CuAAC) reaction (the prototypical “click” reaction) is used as the polymerization step. The AB_2_ monomers bear two azide functionalities and one alkyne functionality, which have been installed onto a 1,3,5 trisubstituted benzene aromatic skeleton. This synthesis has been optimized in terms of its purification strategies, with an eye on its scalability for the potential industrial applications of hyperbranched polymers as viscosity modifiers. By taking advantage of the modularity of the synthesis, we have been able to install short polylactic acid fragments as the spacing units between the complementary reactive azide and alkyne functionalities, aiming to introduce elements of biodegradability into the final products. The hyperbranched polymers have been obtained with good molecular weights and degrees of polymerization and branching, testifying to the effectiveness of the synthetic design. Simple experiments on glass surfaces have highlighted the possibility of conducting the polymerizations and the formation of the hyperbranched polymers directly in thin films at room temperature.

## 1. Introduction

Hyperbranched polymers (HPs) are appealing soft nanomaterials that are used for applications in a wide variety of contexts, spanning catalysis, microelectronics, and nanomedicines, thanks to their unique, branched, dendritic-like architectures that confer their abundant functional groups and intramolecular cavities [1,2,3,4,5,6,7,8,9,10,11,12,13,14]. The low viscosity of HPs, when compared to linear homologues with equivalent molecular weights, is a consequence of their extensive branching. Such a property can be very useful in paint formulations, where the polymeric base constitutes the essential component of the coating system, but it has to be diluted with organic solvents in order to reduce its viscosity and allow for easy handling in its application to a surface [15]. The low viscosity of globular polymers, including HPs, instead of linear polymers with similar compositions and degrees of polymerization, gives the unprecedented advantage of reducing the amount of volatile organic compounds (VOC) in the product for commercialization.

HPs are generally prepared via an effortless one-pot polymerization, which is a striking advantage when compared to soft dendrimer-based materials, which are perfectly branched and globular, but require tedious multistep syntheses and, very often, complicated chromatographic purifications. When compared to dendrimers, HPs are generally achieved with a lower control over the structures and degree of branching (DB < 1) [1]. It was not until the 50s that it was demonstrated that no cross-linking can occur in AB_x_ polycondensation products [16]. In general, the synthesis of HPs can be achieved through three main strategies: (a) a step-growth polymerization of AB_n_ (n ≥ 2) monomers; (b) a self-condensing vinyl polymerization (SCVP) of monomers (AB^#^) containing both a vinyl (A) and an initiating moiety (B^#^); and (c) a multibranching ring-opening polymerization of latent AB_x_ monomers [17]. Thanks to the one-step approach, a variety of HP architectures, such as polyesters [18,19,20], polyethers [21,22,23,24,25], polyurethanes [26,27,28], poly(siloxysilanes) [29,30,31,32,33], polyphenylenes [34], and polyamides [35], have been synthesized. In doing so, chemists have significantly extended their macromolecular architectures beyond traditional linear or ross-linked materials.

The Cu(I)-catalyzed alkyne–azide cycloaddition reaction (CuAAC) is the prototypical and probably most important class of the “click” reaction, which is widely used for bioconjugation [36,37] and the synthesis of macrocycles [38,39], as well as for the grafting and brushing of parental polymers [40,41]. The CuAAC reaction has been used for the construction of HPs, affording polymers with high molecular weights and a high control over the structure and molecular weight distribution (*Ð*), as well as a variety of architectures, thanks to its tolerance toward several functionalities [42]. The first reports on the construction of polytriazole-based HPs through the homopolymerization of AB_2_ monomers were explored by the Voit and Li groups, respectively [43,44]. More recently, Gao and co-workers reported the CuAAC living chain-growth polymerization of AB_2_ monomers for producing HPs with a controlled structure and low *Ð* [45,46,47]. Our group have used a CuAAC polymerization of AB_2_ monomers from 2,2-bis(hydroxymethyl)propionic acid, in which the distance between the polymerizable groups was systematically changed and the effect on the DBs was studied [48].

**Figure 1:**
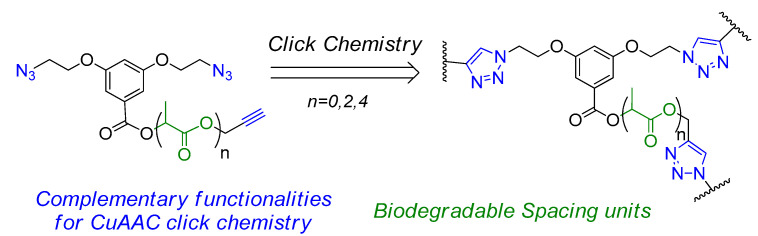
Schematic representation of the key strategy developed in this work.

**Scheme 1:**
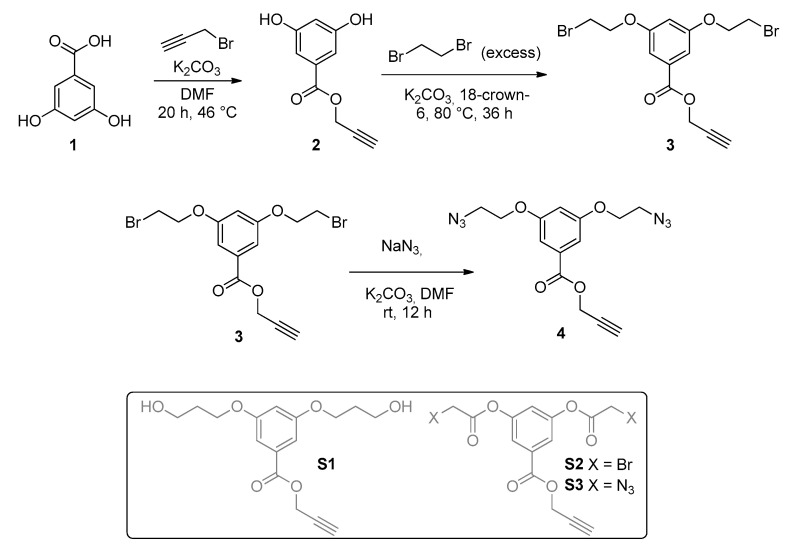
Synthesis of AB_2_ monomer **4**. Bottom: potential AB_2_ monomers **S1** and **S3**, whose synthesis was not achieved (see Supporting Information Scheme S1).

Herein, we report a novel approach to clickable CuAAC monomers based on renewable synthons for obtaining HPs (Figure 1). We approach the introduction of polylactic acid (PLA) chains of variable lengths into the structure of an otherwise rigid, aromatic-based scaffold (Scheme 1), for two main purposes: (a) the introduction of a chemical element of flexibility between the clickable ends; and (b) the introduction of “green” biodegradable fragments, to impart some degrees of biocompatibility onto the chemical structure.

## 2. Results and Discussion

Synthesis of the monomers. Our approach initially explored the synthesis of the rigid, aromatic AB_2_ monomer **4**, starting from commercially available 3,5-dihydroxybenzoic acid **1**.

The synthesis of **4** started with the esterification of compound **1** with propargyl bromide in the presence of potassium carbonate as a base (Scheme 1). The aromatic propargyl ester **2** was obtained with a yield of 61% after simple washing in chloroform, in which compound **2** is not soluble [49]. In order to introduce complementary azide functionalities, we have initially explored several ways for the transformation of the phenolic functionalities of compound **2** (see the Supporting Information Section Figures S1–S13). The alkylation of **2** with 3-chloro-1-propanol on the methyl ester analogue of **2**, using a protocol reported in the literature, failed to produce the product **S1** [50]. An alternative route was thus devised, based on an acylation protocol with bromoacetyl bromide and triethyl amine (TEA) in DCM at room temperature, to produce compound **S2** in a 55% yield. The subsequent nucleophilic substitution with sodium azide, however, following the previously reported protocols for similar compounds [40,51], failed to produce compound **S3**. Thus, we performed the direct alkylation of the phenolic groups using 1,2-dibromoethane in bulk in the presence of potassium carbonate and 18-crown-6 at 80 °C. The corresponding dibromo propargyl ester **3** was obtained in a 34% yield after flash purification. Finally, the reaction with sodium azide and DMF at room temperature afforded the corresponding novel AB_2_ monomer **4** in a 46% yield.

**Scheme 2:**
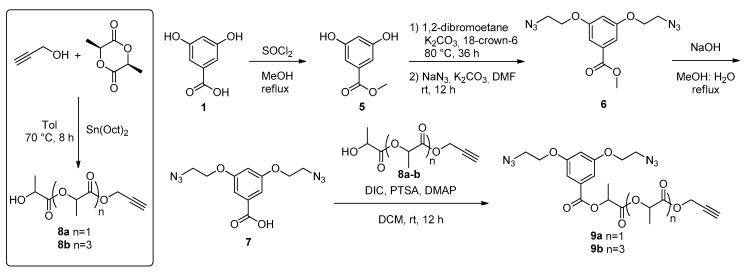
Synthesis of polylactic-containing AB_2_ monomers **9a–b**.

Having established a viable pathway for the efficient synthesis of clickable AB_2_ monomers such as **4**, we focused our efforts on a feasible synthetic route for the extension of our library of monomers and the introduction of PLA chains. The synthesis of compounds **9a–b** is illustrated in Scheme 2.

The esterification reaction of the 3,5-dihydroxybenzoic acid **1**, carried out with thionyl chloride in methanol, produced the pure methyl ester **5** in a 93% yield and followed a protocol from the literature. The alkylation with 1,2-dibromoethane in the previously developed conditions afforded the corresponding dibromo ester in 65% yields, and the replacement of the bromine atoms with azide groups was carried out as before, using sodium azide in DMF as the solvent, to obtain compound **6** in a 99% yield. A saponification using NaOH in MeOH produced the corresponding diazido acid **7** in a 95% yield (Scheme 2).

We performed the ring-opening transesterification of *LL*-lactide with propargyl alcohol as an initiator and tin(II) 2-ethylhexanoate as a catalyst, using a ratio of *LL*-lactide/propargyl alcohol of either 1:1 or 2:1 in the cases of **8a** and **8b**, respectively. In both cases, the products of the ring-opening reaction were purified by precipitation from the dichloromethane into hexane as the nonsolvent. In fact, the ring opening of the *LL*-lactide with propargyl alcohol resulted in enantiomers forming, so that the precise stereochemistry of the polylactic chains was not indicated in the drawing of compound **8**. In any case, since no other elements of chirality were present or incorporated in the following synthetic steps, the formation of diastereoisomers with different properties and stereochemical confusions was avoided. The terminal alkyne functionality, inserted through the use of propargyl alcohol as the initiator, is exploited in the CuAAC click chemistry polymerization reaction.

The coupling reaction between the secondary alcohol group of the oligomers **8a–b** and the carboxylic function of the diazido acid **7**, performed with *N,N’*-diisopropylcarbodiimide (DIC) for reactive coupling in the presence of the salt formed by *p*-toluenesulfonic acid and 4-methylaminopyridine (PTSA-DMAP) as the catalyst, produced the corresponding AB_2_ monomers **9a–b** in 20% and 47% yields, respectively, after purification with column chromatography. The low yields of the coupling, especially in the case of **9a**, could be rationalized by the fact that we observed the presence of by-products generated by the transesterification reaction of the carboxylic acid functionalities onto the reacting PLA chains.

**Figure 2:**
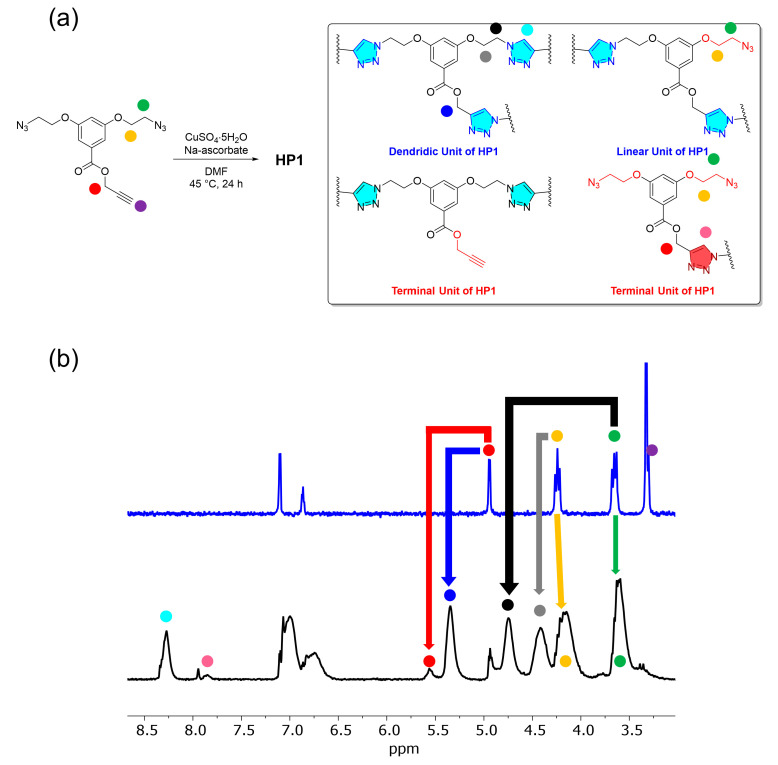
(**a**) Polymerization scheme for monomer **5** and the possible terminal, linear, and dendritic fragments for **HP1**; and (**b**) stacked ^1^H NMR spectra in DMSO-*d*_6_ of monomer **4** (blue line) and hyperbranched polymer **HP1** (black line).

Synthesis of the hyperbranched polymers **HP1-3.** The click polymerization of the AB_2_ monomer **4** was carried out in the presence of catalytic amounts of CuSO_4_·5H_2_O and sodium ascorbate in DMF ([4] = 0.5 M, with a [4]_0_:[Cu]_0_ ratio of 90:1) at 45 °C for 24 h. The complete disappearance of monomer **4** was monitored by TLC and the hyperbranched polymer **HP1** was obtained in a 40% yield after precipitation in hexane. **HP1** was fully characterized, and all its data are reported in the Supporting Information. The hyperbranched structure of **HP1** can, in principle, be composed of a mixture of the dendritic, linear, and terminal units represented in Figure 2a. The comparison of the ^1^H NMR spectra of the purified, precipitated polymer **HP1** with the starting AB_2_ monomer **4** is shown in Figure 2b.

The ^1^H-NMR spectrum of **HP1** testified to the successful occurrence of the polymerization reaction, given that: (a) the proton resonances were broadened, due to the formation of a macromolecular structure in which all the repeating units were slightly different from each other; (b) there was an appearance of the triazole proton resonances at 8.3 ppm (cyan marker) in the polymer ^1^H-NMR spectrum (black line); and (c) there was a disappearance of the terminal alkyne groups of the monomer, such as the -CH_2_ groups at 4.8 ppm (dark blue dots), and of the terminal alkyne proton signal at ca. 3.5 ppm (pink). The deshielding effects on the other CH_2_ groups were also diagnostic: the newly triazole moieties in **HP1** caused a downshift in the CH_2_ protons at 5.3 ppm (blue marker), 3.7 ppm (black marker), and 4.4 ppm (gray marker) compared to those of monomer **4**, which were centered at 4.9 ppm (red marker), 4.2 (orange marker), and 3.5 ppm (green marker), respectively. The remaining broadened signals at 4.2 ppm and 3.6 ppm corresponded to the protons of the linear units in **HP1**. We attributed the small signals at 7.8 ppm and 5.6 ppm to the azide-containing terminal units of the hyperbranched structure, according to the literature [52]. In fact, only unreacted azido groups could be detected in the purified **HP1**. This evidence is in accordance with the FTIR spectra (see Supporting Information), in which the typical C-H stretching of triple bonds (ca. 3300 cm^−1^) was not observed, while the stretching band of the azido group at ca. 2100 cm^−1^ was detectable. Such an observation is supported when also considering the ^13^C-NMR spectra of **HP1**, in which a diagnostic signal at ca. 50 ppm, related to the CH_2_N_3_ carbon resonance, was present.

The degree of the branching (DB) of **HP1** was determined using the unequivocally established proton peaks of the dendritic units (D) and linear units, following the equation DB = 1/(1 + 0.5 × (L/D)) [53]. The value obtained for **HP1** was 0.43. The relevant data for all the hyperbranched polymers are reported in Table 1.

The same conditions for the CuAAC polymerization of the aromatic diazido propargyl ester **4** were used for monomers **9a–b**. After a TLC monitoring of the full conversion of the monomer, the crude products were precipitated in hexane, obtaining the hyperbranched polymers **HP2** and **HP3** as white powders, respectively, with yields of 40%, and 56%, without any significant differences in their aspects when compared to **HP1**. This observation suggested that the additional PLA chains in **HP2** and **HP3** did not significantly chelate the colored Cu catalyst. ^1^H NMR and GPC analyses of the crude reaction mixtures in either **HP1**, **HP2,** or **HP3** did not show the presence of low molecular weight compounds to be potentially associated with the intramolecularly cyclized products.

**Figure 3:**
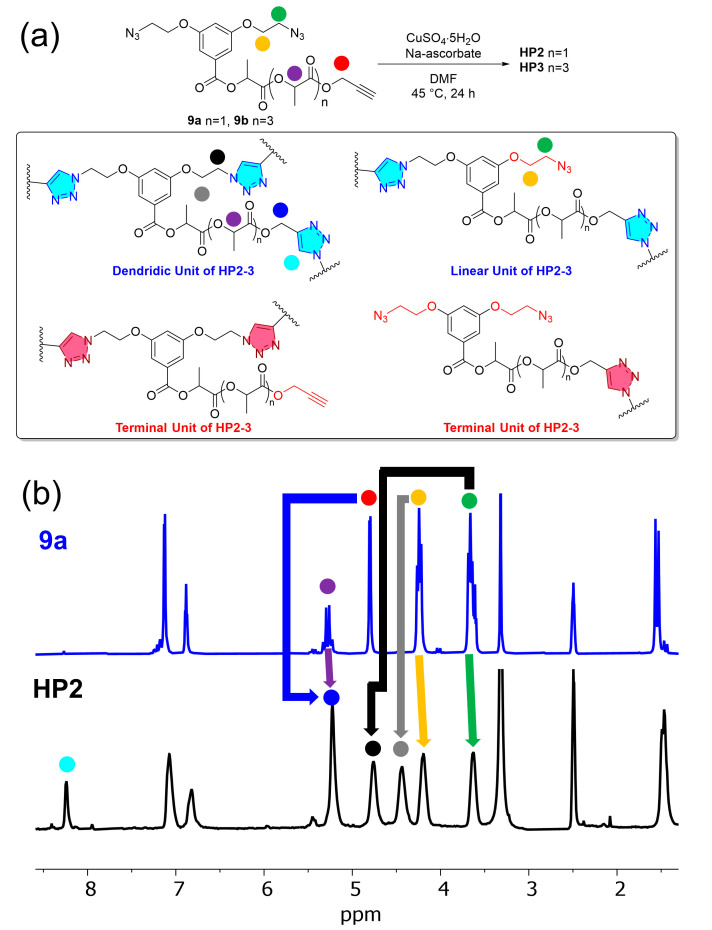
(**a**) Polymerization scheme of monomers **9a–b**, and (**b**) stacked ^1^H NMR spectra in DMSO-*d*_6_ of monomer **9a** (blue line) and hyperbranched polymer **HP2** (black line).

A comparison between the ^1^H-NMR spectra in the DMSO-*d*_6_ of the AB_2_ monomer **9a** and the hyperbranched polymer **HP2** after precipitation is shown in Figure 3, whereas the stacked ^1^H NMR spectra of **9b** vs. **HP3** are shown in the Supporting Information. The comparison between the spectra in Figure 3 testifies to the occurrence of the polymerization reaction. All the diagnostic signals shifted in a similar manner to that which was described for **HP1**, with only a few differences: the CH_2_ proton resonances neighboring the newly generated triazole moieties of the dendritic units were superimposed with the α-CH proton resonances of the lactic ester units, and both the possible terminal units were not present in this case, and in the case of **HP3** (see Supporting Information).

Table 1 summarizes the main properties of the discussed HPs.

A gel permeation chromatography (GPC) analysis was performed in THF to determine the average molecular weights and molecular weight distributions of the hyperbranched polymers, which were calculated against a calibration curve built using narrow polydispersity linear polystyrene standards. The results therefore have to be taken as a qualitative comparison between the **HPs**, since they do not take into account the well-documented differences between the hydrodynamic radii of the HPs, with respect to their structurally related linear polymers with equivalent degrees of polymerizations [1]. However, an increase in *M*_n_ and the degree of polymerizations, when passing from **HP1** to **HP3**, was noticeable, presumably as a result of the enhanced flexibility of the overall monomers, achieved by adding PLA chains between the reactive azide and alkyne functionalities. On the other hand, the DB seemed to decrease as the linear polymerization route became advantageous, presumably because the added flexibility of the longer PLA chain counterbalanced its steric hindrance within its random coil conformation.

**Click polymerization on surfaces.** In order to verify the applicability of the click reaction protocol directly onto a surface, we performed experiments using the AB_2_ monomer **4** in thin film formulations. We preliminarily tested the filmability of monomer **4** and the corresponding **HP1**. As expected, monomer **4**, as a low molecular weight molecule, did not form homogeneous solutions when drop-casted onto glass surfaces, but instead, **HP1** showed good filmability properties and formed a homogeneous film when drop-casted onto a glass slide, as visually inspected by an optical microscope. The nonoptimal filmability of monomer **4** ruled out the possibility of a further analysis and comparison between the films using contact angle measurements. In a further experiment, a reaction mixture containing AB_2_ monomer **4**, ascorbic acid, and CuSO_4_·5H_2_O in DMF was drop-casted from the DMF (100 µL) onto a glass slide, where it showed the formation of a homogeneous film after 16 h at room temperature. A control experiment performed in the same conditions but lacking the Cu catalyst did not show the formation of a homogeneous film (see Supporting Information). The occurrence of click hyperbranched polymerization was confirmed by the FTIR and ^1^H NMR spectroscopies.

## 3. Materials and Methods

All the commercially available compounds and reaction solvents were purchased from Merck, TCI, Fluorochem, and used as received. Compound **5** [54] and catalyst *p*-toluenesulfonate dimethylaminopyridinium salt (PTSA-DMAP) [55,56] were synthesized following a reported procedure. Dry dichloromethane was obtained through the distillation of the solvent in the presence of calcium hydride. The thin-layer chromatography was performed on commercially available TLC Silica gel 60 F_254_ plates. The column chromatography was carried out using silica gel (pore size 60 Å, 230–400 mesh). The ^1^H and ^13^C-NMR spectra were recorded on Bruker AX200 or AMX300 instruments and calibrated with the solvent residual proton signal or tetramethyl silane. The MS spectra were obtained with an Agilent ion trap mass spectrometer equipped with an ESI ion source. The IR spectra were recorded on an FTIR spectrophotometer equipped with a diffuse reflectance accessory, using KBr powder as the inert support. SEC was carried out on a Waters system equipped with an RI detector. Narrow polydispersity polystyrene standards were used for the calibration curve and the mobile phase was THF stabilized with BHT (1 mL/min, 40 °C). A set of two universal columns (Styragel 4E and 5E) in series were used. The samples were prepared by solubilizing the hyperbranched polymers in THF and were prefiltered on 0.45 μm PTFE filters before injection.

**Compound 2.** K_2_CO_3_ (17.94 g, 130 mmol) was added to a solution of 3,5-dihydroxybenzoic acid **1** (20 g, 130 mmol) in DMF (50 mL); the propargyl bromide (15.9 mL, 143 mmol) was added dropwise and the reaction mixture was stirred for 20 h at 45 °C. The solvent was removed, water (100 mL) was added, and the residue was extracted with EtOAc (4 × 120 mL). The organic phase was washed with an aqueous solution of NH_4_Cl 1 M (5 × 200 mL), dried over Na_2_SO_4_, filtered, and concentrated under reduced pressure. The crude product was purified, washed with CHCl_3_, filtered, and dried under vacuum, to provide a pale yellow solid corresponding to the pure compound **2** (15.16 g, 61%).

^1^H-NMR (DMSO-*d*_6_, 200 MHz): δ (ppm) = 9.67 (s, 2H, -O*H*), 6.82-6.81 (d, 2H, -Ph), 6.46-6.45-6.44 (t, 1H, -Ph), 4.89-4.88 (d, 2H, -OC*H_2_*CCH), and 3.60-3.59-3.58 (t, 1H, -C*H*). ^13^C-NMR (DMSO-*d6*, 300 MHz): δ (ppm) = 164.82 (-*C*OOCH_2_CCH), 158.46 (2C, HO-*C*H=), 130.45 (-OOC-*C*H=), 107.38 (HO-CH=*C*H=CH-OH), 107.00 (2C, HO-CH=*C*H=CH-COO-), 78.28 (-OCH_2_*C*CH), 77.54 (-*C*H), and 52.16 (-O*C*H_2_CCH). ESI-MS (MeOH): *m/z* 191 [M - H]^−^, 383 [2M - H]^−^.

**Compound 3**. The propargyl ester **2** (2 g, 10.4 mmol) was dissolved in 1,2-dibromoethane (21 mL) and K_2_CO_3_ (3.6 g, 26 mmol) was added, followed by 18-crown-6 (165 mg, 0.624 mmol); the suspension was stirred for 36 h at 80 °C. The reaction mixture was cooled and filtered on büchner and washed with CHCl_3_: the solution was evaporated under reduced pressure and the residue was purified by column chromatography (silica gel, R_f_ = 0.51, hexane:EtOAc, 8:2, *v*/*v*) to afford product **3** as a white solid (1.44 g, 34%). ^1^H-NMR (CDCl_3_, 200 MHz): δ (ppm) = 7.25-7.24 (d, 2H, -Ph), 6.74-6.73-6.72 (t, 1H, -Ph), 4.93-4.92 (d, 2H, -OC*H_2_*CCH), 4.37-4.33-4.30 (t, 4H, BrCH_2_C*H_2_*O-), 3.69-3.66-3.63 (t, 4H, BrC*H_2_*CH_2_O-), and 2.55-2.53-2.52 (t, 1H, -C*H*). ^13^C-NMR (CDCl_3_, 300 MHz): δ (ppm) = 165.10 (-*C*OOCH_2_CCH), 159.11 (2C, BrCH_2_CH_2_O-*C*H=), 131.39 (-OOC-*C*H=), 108.55 (2C, -O-CH=*C*H=CH-COO-), 107.40 (-O-CH=*C*H=CH-O-), 77.33 (-OCH_2_*C*CH), 75.10 (-*C*H), 68.02 (2C, BrCH_2_*C*H_2_O-), 52.61 (-O*C*H_2_CCH), and 28.72 (Br*C*H_2_CH_2_O-). ESI-MS (MeOH): *m/z* 429 [M + Na]^+^, 834 [2M + Na]^+^.

**Compound 4**. NaN_3_ (679 mg, 10.4 mmol) was added to a solution of compound **3** (1.41 g, 3.48 mmol) in dry DMF (25 mL) and the suspension was stirred overnight at room temperature. An aqueous solution of NH_4_Cl 1 M (15 mL) was added to the reaction mixture and the product was extracted with CH_2_Cl_2_ (3 × 30 mL). The organic phase was washed with an aqueous solution of NH_4_Cl 1 M (5 × 70 mL) and then dried over Na_2_SO_4_ and filtered. The solvent was evaporated under reduced pressure. The residue was purified by column chromatography (silica gel, R_f_ = 0.26, hexane:EtOAc, 8:2, *v*/*v*) to afford product **4** as a white solid (535 mg, 46%). ^1^H-NMR (CDCl_3_, 200 MHz): δ (ppm) = 7.26–7.25 (d, 2H, -Ph), 6.74-6.73-6.72 (t, 1H, -Ph), 4.93-4.92 (d, 2H, -OC*H_2_*CCH), 4.21-4.19–4.16 (t, 4H, N_3_CH_2_C*H*_2_O-), 3.66-3.62-3.60 (t, 4H, N_3_C*H_2_*CH_2_O-), and 2.54-2.53-2.52 (t, 1H, -C*H*). ^1^H-NMR (DMSO-*d6*, 200 MHz): δ (ppm) = 7.11-7.10 (d, 2H, -Ph), 6.87-6.86-6.85 (t, 1H, -Ph), 4.96-4.94 (d, 2H, -OC*H_2_*CCH), 4.27-4.24-4.22 (t, 4H, N_3_CH_2_C*H_2_*O-), and 3.68-3.65-3.63 (t, 5H, N_3_C*H_2_*CH_2_O- and -C*H*). ^13^C-NMR (CDCl_3_, 300 MHz): δ (ppm) = 165.12 (-*C*OOCH_2_CCH), 159.20 (2C, N_3_CH_2_CH_2_O-*C*H=), 131.37 (-OOC-*C*H=), 109.33 (2C, -O-CH=*C*H=CH-COO-), 107.26 (-O-CH=*C*H=CH-O-), 77.41 (-OCH_2_*C*CH), 75.08 (-*C*H), 67.18 (2C, N_3_CH_2_*C*H_2_O-), 52.59 (-O*C*H_2_CCH), and 49.94 (N_3_*C*H_2_CH_2_O-). ESI-MS (MeOH): *m/z* 353 [M + Na]^+^, 683 [2M + Na]^+^. IR (cm^−1^): 1716.9 (C=O str), 2109.9 (N_3_ str), and 3277.6 (CCH str).

**Compound 6.** The methyl ester **5** (2 g, 11.9 mmol) was dissolved in 1,2-dibromoethane (24 mL) and K_2_CO_3_ (4.11 g, 29.7 mmol) was added, followed by 18-crown-6 (189 mg, 0.714 mmol). The reaction mixture was stirred at 80 °C for 36 h. The reaction mixture was cooled and filtered on büchner, washed with CHCl_3_, and the solvent was removed under reduced pressure. The crude reaction product was purified by column chromatography (SiO_2_, hexane/EtOAc 8:2, R_f_ = 0.53) to give an intermediate as a white solid (2.96 g, 65%). NaN_3_ (1.51 g, 23.2 mmol) was added to a solution of the intermediate in dry DMF. The reaction mixture was stirred at room temperature overnight. After TLC monitoring, a 1 M aqueous solution of NH_4_Cl (30 mL) was added to the reaction mixture and the product was extracted with DCM (3 × 60 mL). The organic phase was washed with a 1 M aqueous solution of NH_4_Cl (5 × 150 mL) and then dried over Na_2_SO_4_ and filtered. The solvent was evaporated under reduced pressure to afford product **6** as a colorless oil (2.34 g, 99%). ^1^H NMR (CDCl_3_, 200 MHz) *δ* (ppm): 7.24 (d, 2H, -Ph), 6.71 (t, 1H, -Ph), 4.19 (t, 4H, N_3_CH_2_CH_2_O-), 3.92 (s, 3H, -OCH_3_), and 3.62 (t, 4H, N_3_CH_2_CH_2_O-).

**Compound 7.** NaOH (1.76 g, 44 mmol) in H_2_O (5.8 mL) was added to a solution of compound **6** (2.27 g, 7.42 mmol) in MeOH (11.7 mL) and the reaction mixture was stirred overnight at room temperature. The MeOH was removed under reduced pressure, water (20 mL) was added, and the aqueous phase was slowly acidified with HCl 2 N until the precipitation of a white solid. The product was extracted with DCM (3 × 40 mL) and the organic phase was dried over Na_2_SO_4_ and filtered. The solvent was evaporated under reduced pressure to produce compound **7** as a white solid (2.06 g, 95%). ^1^H NMR (CDCl_3_, 200 MHz) *δ* (ppm): 7.31 (d, 2H, -Ph), 6.78 (t, 1H, -Ph), 4.22 (t, 4H, N_3_CH_2_CH_2_O-), and 3.65 (t, 4H, N_3_CH_2_CH_2_O-).

**Compound 8a.** *LL*-lactide (2 g, 13.9 mmol) and dry toluene (9 mL) were charged in a Schlenk flask, which was capped with rubber septa and bubbled with nitrogen gas; a solution of Sn(oct)_2_ (3.14 mL, 9.71 mmol) and propargyl alcohol (1.7 mL, 29.1 mmol) in dry toluene (5 mL) was added and the reaction mixture was stirred for 7 h at 70 °C. The suspension was cooled and filtered on büchner. The solution was evaporated under reduced pressure to remove the toluene. The residue was washed several times with hexane to produce a pale yellow oil, corresponding to product **8a** (1.67 g, 75%). ^1^H-NMR (CDCl_3_, 200 MHz): δ (ppm) = 5.25-5.22-5.18-5.15 (q, 1H, -COC(CH_3_)*H*-OH), 4.74-4.73-4.72 (t, 2H, -OC*H_2_*CCH), 4.41-4.37-4.34-4.30 (q, 1H, -COC(CH_3_)*H*-OCO-), 2.51 (t, 1H, -C*H*), and 1.56 (m, 6H, -C*H_3_*).

**Compound 8b.** *LL*-lactide (2 g, 13.9 mmol) and dry toluene (9 mL) were charged in a Schlenk flask, which was capped with rubber septa and bubbled with nitrogen gas; a solution of Sn(oct)_2_ (764 µL, 2.36 mmol) and propargyl alcohol (404 µL, 6.94 mmol) in dry toluene (5 mL) was added and the reaction mixture was stirred for 7 h at 70 °C. The suspension was cooled and filtered on büchner. The solution was evaporated under reduced pressure to remove the toluene. The residue was dissolved in CH_2_Cl_2_ (10 mL) and precipitated in hexane (150 mL): a white viscous precipitate formed, which was collected and dried under vacuum, to obtain the pure compound **8b** (1.15 g, 55%). ^1^H-NMR (CDCl_3_, 200 MHz): δ (ppm) = 5.25-5.21-5.17-5.13 (q, 3.53H, -COC(CH_3_)*H*-OCO-), 4.75-4.74-4.72 (t, 2H, -OC*H_2_*CCH), 4.42-4.39-4.35-4.32 (q, 1H, -COC(CH_3_)*H*-OH), 2.52-2.51-2.50 (t, 1H, -C*H*), and 1.56 (m, 13.59H, -C*H_3_*).

**Compound 9a.** Compound **8a** (630 mg, 3.15 mmol), PTSA-DMAP (1.85 g, 6.29 mmol), and dry CH_2_Cl_2_ (6 mL) were charged in a Schlenk flask, which was capped with rubber septa and bubbled with nitrogen gas; compound **7** (920 mg, 3.15 mmol) was added, followed by DIC (1.46 mL, 9.44 mmol), and the reaction mixture was stirred at room temperature overnight. Water (9 mL) was added, the product was extracted with CH_2_Cl_2_ (3 × 10 mL), and the organic phase was dried over Na_2_SO_4_, filtered, and evaporated under reduced pressure. The residue was purified by column chromatography (silica gel, R_f_ = 0.2, hexane:EtOAc, 8:2, *v*/*v*) to afford product **9a** as a colorless oil (299 mg, 20%). ^1^H-NMR (CDCl_3_, 200 MHz): δ (ppm) = 7.27-7.26 (d, 2H, -Ph), 6.74-6.73-6.72 (t, 1H, -Ph), 5.40-5.37-5.33-5.29 (q, 1H, -C(CH_3_)*H*-), 5.29-5.25-5.22-5.18 (q, 1H, -C(CH_3_)*H*-), 4.75-4.74-4.73 (t, 2H, -OC*H_2_*CCH), 4.21-4.18-4.16 (t, 4H, N_3_CH_2_C*H_2_*O-), 3.65-3.62-3.60 (t, 4H, N_3_C*H_2_*CH_2_O-), 2.52-2.51-2.50 (t, 1H, -C*H*), 1.74-1.70 (d, 3H, -C*H_3_*), and 1.60-1.57 (d, 3H, -C*H_3_*). ^1^H-NMR (DMSO-*d6*, 200 MHz): δ (ppm) = 7.13-7.12 (d, 2H, -Ph), 6.89-6.88-6.87 (t, 1H, -Ph), 5.37-5.33-5.30-5.26 (q, 1H, -C(CH_3_)*H*-), 5.26-5.22-5.19-5.15 (q, 1H, -C(CH_3_)*H*-), 4.79-4.77 (d, 2H, -OC*H_2_*CCH), 4.26-4.24-4.22 (t, 4H, N_3_CH_2_C*H_2_*O-), 3.68-3.66-3.64 (t, 5H, N_3_C*H_2_*CH_2_O- and -C*H*), 1.61-1.58 (d, 3H, -C*H_3_*), and 1.48-1.44 (d, 3H, -C*H_3_*). ^13^C-NMR (CDCl_3_, 300 MHz): δ (ppm) = 169.93 (-*C*OC(CH_3_)H-O-), 169.35 (-*C*OC(CH_3_)H-O-), 165.30 (-*C*OOC(CH_3_)H-), 159.19 (2C, N_3_CH_2_CH_2_O-*C*H=), 131.30 (-OOC-*C*H=), 108.42 (2C, -O-CH=*C*H=CH-COO-), 107.30 (-O-CH=*C*H=CH-O-), 77.36 (-OCH_2_*C*CH), 75.47 (-*C*H propargyl), 69.02 (-CO*C*(CH_3_)H-O-), 68.85 (-CO*C*(CH_3_)H-O-), 67.16 (2C, N_3_CH_2_*C*H_2_O-), 52.76 (-O*C*H_2_CCH), 49.94 (N_3_*C*H_2_CH_2_O-), 16.76 (-COC(*C*H_3_)H-O-), and 16.56 (-COC(*C*H_3_)H-O-). ESI-MS (MeOH): *m/z* 497 [M + Na]^+^, 971 [2M + Na]^+^. IR(cm^−1^): 1756.1 (C=O str), 2108.1 (N_3_ str), and 3290.6 (CCH str).

**Compound 9b.** Compound **8b** (320 mg, 0.836 mmol), PTSA-DMAP (492 mg, 1.67 mmol), and dry CH_2_Cl_2_ (2 mL) were charged in a Schlenk flask, which was capped with rubber septa and bubbled with nitrogen gas; compound **7** (244 mg, 0.836 mmol) was added, followed by DIC (389 µL, 2.51 mmol), and the reaction mixture was stirred at room temperature overnight. Water (20 mL) was added, the product was extracted with CH_2_Cl_2_ (3 × 20 mL), and the organic phase was dried over Na_2_SO_4_, filtered, and evaporated under reduced pressure. The residue was purified by a short chromatographic column (silica gel, hexane:EtOAc, 7:3, **v*/*v**) to afford product **9b** as a colorless oil (257 mg, 47%). ^1^H-NMR (CDCl_3_, 200 MHz): δ (ppm) = 7.27-7.26 (d, 2H, -Ph), 6.74-6.73-6.72 (t, 1H, -Ph), 5.40-5.37-5.33-5.30 (q, 1H, -C(CH_3_)*H*-), 5.24-5.20-5.16-5.13 (q, 3.53H, -C(CH_3_)*H*-), 4.75-4.74-4.72 (t, 2H, -OC*H_2_*CCH), 4.21-4.18-4.16 (t, 4H, N_3_CH_2_C*H_2_*O-), 3.64-3.62-3.60 (t, 4H, N_3_C*H_2_*CH_2_O-), 2.52-2.51-2.50 (t, 1H, -C*H*), and 1.64 (m, 13.59H, -C*H_3_*). ^1^H-NMR (DMSO-*d6*, 200 MHz): δ (ppm) = 7.13-7.12 (d, 2H, -Ph), 6.89-6.88-6.87 (t, 1H, -Ph), 5.40-5.37-5.33-5.30 (q, 1H, -C(CH_3_)*H*-), 5.26-5.22-5.19-5.15 (q, 3.53H, -C(CH_3_)*H*-), 4.78-4.77 (d, 2H, -OC*H_2_*CCH), 4.26-4.24-4.22 (t, 4H, N_3_CH_2_C*H_2_*O-), 3.68-3.66-3.64 (t, 5H, N_3_C*H_2_*CH_2_O- and -C*H*), 1.59-1.56 (d, 3H, -C*H_3_*), and 1.48-1.45 (d, 10.59H, -C*H_3_*).

**Polymers HP1-3.***Selected example of CuAAC polymerization for HP1. *A flame-dried Schlenk flask was charged, under a nitrogen atmosphere, with monomer **4** (150 mg, 0.454 mmol), CuSO_4_·5H_2_O (1.2 mg, 5 µmol), and dry DMF (922 µL). The reaction mixture was degassed with nitrogen for 40 min and ascorbic acid (4.5 mg, 25.4 µmol) was added and warmed with a thermostatic oil bath at 45 °C. After 24 h, the reaction mixture was cooled to room temperature and the solvent was removed under reduced pressure. The crude reaction product was dissolved in THF (1 mL) and precipitated in hexane (12 mL) to produce the desired polymer.

**HP1.** From compound **3**, a white solid was produced (60 mg, 40%). ^1^H-NMR (DMSO-*d_6_*, 200 MHz) δ (ppm): 8.27 (s, 1H, CH triazole), 7.03 (d, 2H, -Ph), 6.84 (t, 1H, -Ph), 5.35 (s, 2H, -COOCH_2_ArH), 4.75 (s, 2H, -OCH_2_CH_2_ArH), 4.42 (s, 2H, -OCH_2_CH_2_ArH), 4.22 (t, 4H, N_3_CH_2_CH_2_O-), and 3.63 (t, 4H, N_3_CH_2_CH_2_O-).

**HP2.** From compound **9a**, a white solid was produced (71 mg, 38%). ^1^H NMR (DMSO-*d*_6_, 200 MHz) *δ* (ppm): 8.24 (s, 1H, CH triazole), 7.09 (d, 2H, -Ph), 6.82 (t, 1H, -Ph), 5.23 (s, 3H, -C(CH_3_)H- and -COOCH_2_ArH), 4.76 (s, 2H, -OCH_2_CH_2_ArH), 4.44 (s, 2H, -OCH_2_CH_2_ArH), 4.19 (s, 4H, N_3_CH_2_CH_2_O-), 3.63 (s, 4H, N_3_CH_2_CH_2_O-), and 1.49-1.47 (d, 3H, -CH_3_).

**HP3.** From compound **9b**, a white solid was produced (100 mg, 56%). ^1^H NMR (DMSO-*d_6_*, 200 MHz) *δ* (ppm): 8.26 (s, 1H, CH triazole), 7.10 (d, 2H, -Ph), 6.86 (t, 1H, -Ph), 5.20 (broad s, 6H, -C(CH_3_)H- and -COOCH_2_ArH), 4.77 (s, 2H, -OCH_2_CH_2_ArH), 4.46 (s, 2H, -OCH_2_CH_2_ArH), 4.22 (s, 4H, N_3_CH_2_CH_2_O-), 3.65 (s, 4H, N_3_CH_2_CH_2_O-), 1.58 (d, 3H, -CH_3_), and 1.46 (d, 10H, -CH_3_).

## 4. Conclusions

Through CuAAC click chemistry, we demonstrated the possibility of constructing new AB_2_ monomers that were suitable for polymerization. The synthesis was designed to be modular, so that the introduction of short PLA fragments as the spacing units between the complementary reactive azide and alkyne functionalities could be successfully optimized. In such a way, elements of biodegradability were indeed introduced into the final products. All the monomers were able to polymerize yielding structures with significant degrees of polymerization and branching. Simple experiments on glass surfaces highlighted the possibility of conducting this polymerization directly in thin films in an open environment. The proposed synthetic pathway for the synthesis of the AB_2_ monomers was flexible and adaptable, so that other molecular fragments or oligomers, in order to tune the materials’ properties, could, in principle, be inserted between the alkyne and azide reactive units. Future work will focus on increasing the sustainability and scalability of the syntheses of these and related systems, and on an improved design for obtaining higher DBs that are more useful for industrial applications.

## Figures and Tables

**Table 1:** Main characteristics of hyperbranched polymers **HP1-3** ^1^.

Entry	HP	Yield ^2^	*M*_n_	*M*_w_	*DP * ^3^	*DB * ^4^
1	**HP1**	40	1700 ^5^	2600 ^5^	5	0.43
2	**HP2**	38	3500 ^5^	10,400 ^5^	7	0.39
3	**HP3**	56	4800	9500	8	0.33

^1^ Reaction time: 24 h. [AB_2_]_0_ = 0.5 M in DMF, 45 C. ^2^ After precipitation by purification in hexane. ^3^ Degree of polymerization. Obtained dividing *M*_n_ by the mass of the starting monomer. ^4^ Degree of branching. See text for details. ^5^ Bimodal distributions observed in the GPC trace.

## Data Availability

The data presented in this study are available in the Supporting Information Section.

## Supplementary Materials

The following supporting information can be downloaded at: https://www.mdpi.com/article/10.3390/ijms24087620/s1.
