# Supplementary material for: Polylactic-Containing Hyperbranched Polymers through the CuAAC Polymerization of Aromatic AB2 Monomers
Source: Int J Mol Sci. 2023 Apr 21;24(8):7620. doi: 10.3390/ijms24087620 (PMC10145021; doi:10.3390/ijms24087620)
Supplement: Supplementary file 1 [file ijms-24-07620-s001.zip › ijms-2331913-supplementary.pdf]

Electronic Supporting Information for:

Polylactic-Containing Hyperbranched Polymers through the CuAAC Polymerization of Aromatic AB_2 Monomers

Aurora Pacini, Andrea Nitti, Marcello Vitale and Dario Pasini

1	Attempted synthesis of monomers S1-4	2
2	Characterization of HP1	3
3	Characterization of HP2	7
4	Characterization of HP3	10
5	Thin Film Experiments	13
6	Characterization of New Compounds	16

1. Attempted synthesis of monomers S1-4

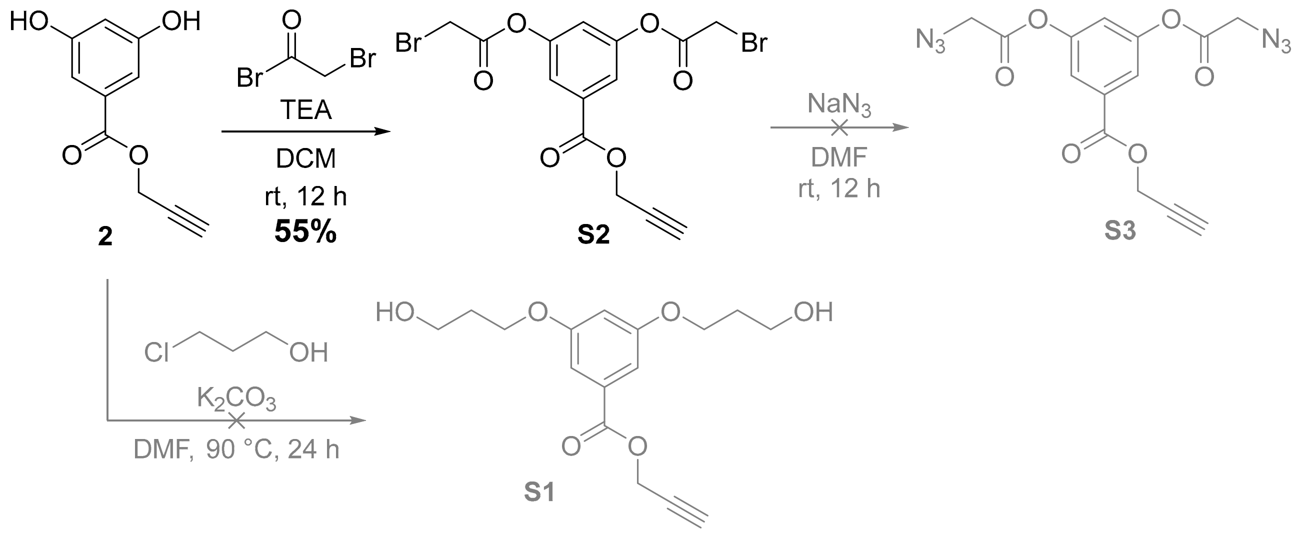

Scheme S1. Attempted synthesis of compound **S1-S3**.

Attempted procedure for the synthesis of compound S1. K_2CO_3 (4.73 g, 34.2 mmol) was added to a solution of the propargyl ester (**14**) (1 g, 5.2 mmol) in DMF (20 mL); 3-chloro-1-propanol (887 µL, 10.6 mmol) was added followed by KI (some pellets) and the reaction mixture was stirred for 24 hours, at 90°C. The suspension was poured into the water (50 mL) and extracted with $CHCl_3$ (3 x 70 mL). The organic phase was washed with water (100 mL), brine (100 mL) and then dried over Na_2SO_4, filtered and evaporated under reduced pressure. ^{1}H-NMR spectrum was recorded and the desired product was not observed.

Compound S2. The propargyl ester **2** (500 mg, 2.6 mmol) was dissolved in DCM (10 mL). TEA (798 µL, 5.72 mmol) was added at 0°C followed by bromoacetyl bromide (907 µL, 10.4 mmol). The mixture was stirred at room temperature overnight, then they was diluted with DCM (10 mL) and washed with a saturated solution of $NaHCO_3$ (3 x 15 mL). The organic phase was dried over Na_2SO_4, filtered and concentrated under reduced pressure. The reaction crude was purified by column chromatography (SiO_2, , Hexane/EtOAc 8:2, R_f = 0.32) to afford the product **S3** as a colourless oil (626 mg, 55 %). ^{1}H-NMR ($CDCl_3$, 200 MHz): δ (ppm) = 7.79-7.78 (d, 2H, -Ph), 7.28-7.27-7.26 (t, 1H, -Ph), 4.96-4.95 (d, 2H, -OC$\underline{H_2}$CCH), 4.08 (s, 4H, -OCOC$\underline{H_2}$Br), 2.57-2.56-2.55 (t, 1H, -C$\underline{H}$). ^{13}C-NMR ($CDCl_3$, 300 MHz): δ (ppm) = 165.03 (2C, -O$\underline{C}$OCH$_2$Br), 163.59 (-$\underline{C}$OOCH$_2$CCH), 150.59 (2C, BrCH$_2$COO-$\underline{C}$H=), 131.80 (-OOC-$\underline{C}$H=), 120.50 (2C, -O-CH=$\underline{C}$H=CH-COO-), 119.57 (-O-CH=$\underline{C}$H=CH-O-), 77.33 (-OCH$_2\underline{C}$CH), 75.47 (-$\underline{C}$H), 53.00 (-O$\underline{C}$H$_2$CCH), 24.85 (2C, -OCO$\underline{C}$H$_2$Br). ESI-MS (MeOH): *m/z* 457 $[M + Na]^+$, 473 $[M + K]^+$, 891 $[2M + Na]^+$, 906 $[2M + K]^+$.

Attempted procedure for the synthesis of compound S3. NaN_3 (135 mg, 2.07 mmol) was added to a solution of compound **S3** (300 mg, 0.691 mmol) in dry DMF (5 mL) and the suspension was stirred at room temperature overnight. An aqueous solution of NH_4Cl 1 M (5 mL) was added to the reaction mixture and the residue was extracted with DCM (3 x 7 mL). The organic phase was washed with an aqueous solution of NH_4Cl 1 M (5 x 15 mL) and then dried over Na_2SO_4, filtered and the solvent was evaporated under reduced pressure. The desired product was not observed in the ^{1}H-NMR spectrum of the crude reaction mixture.

2. Characterization of HP1

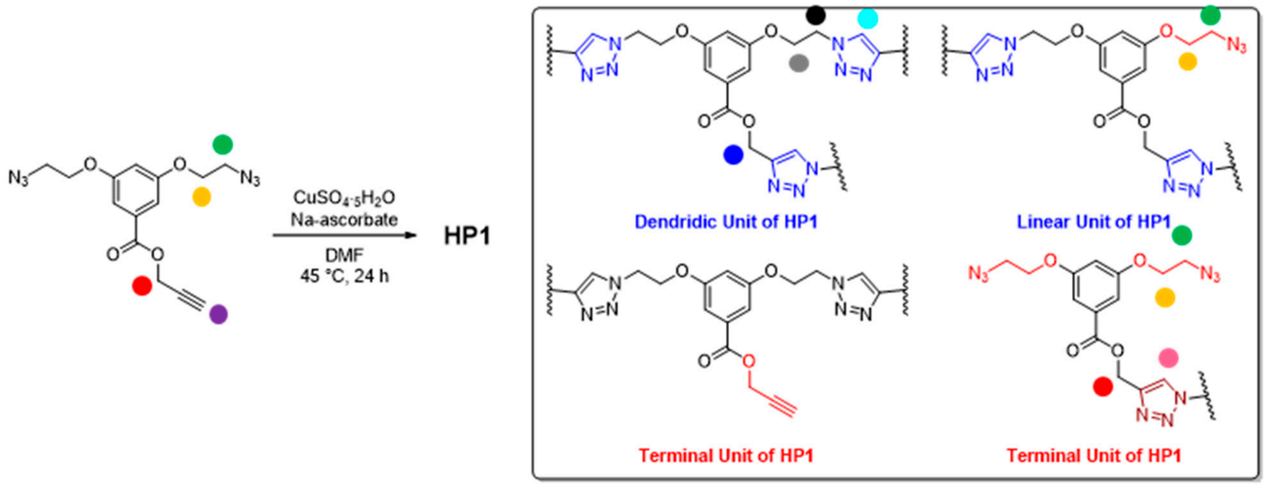

In Figure S1 and S2 we report the ^{1}H NMR spectra of compound **4** and polymer **HP1**, while in Figure S3 we report the ^{13}C NMR and HSQC spectra of **HP1** in DMSO-d_6. In Figure S4 we report the FTIR spectra of compound **4** and **HP1**. The DB was obtained according to following equation:

$$DB = \frac{1}{1 + 0.5(L/D)}$$

Where L and D corresponding to integrals of linear (L) and dendridic (D) units respectively.

By using the relative integration in the ^{1}H NMR spectra of Figure S1 we obtain L = 2.82 (CH_2 green dots Linear +Terminal) – 0.39/2 (red dots 2X CH_2 Terminal) = 2.625, and D = (CH_2 blue dots Dendritic) = 1.95.

DB = 0.43

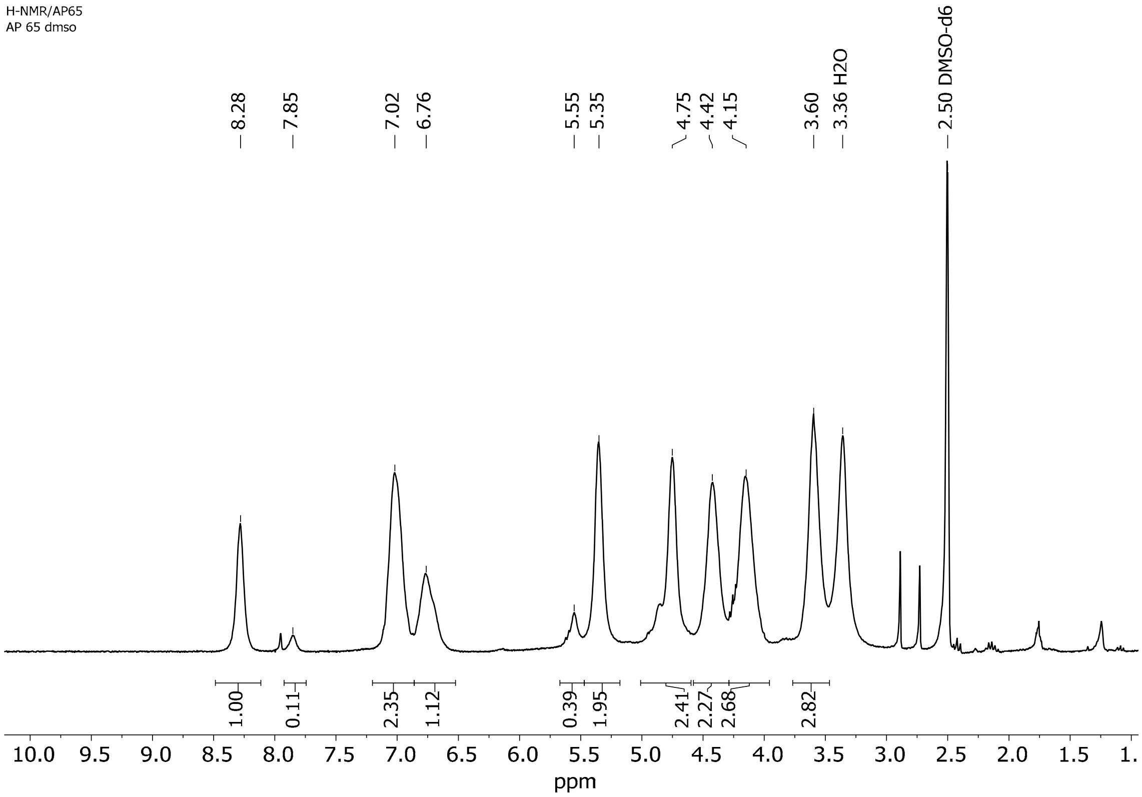

Figure S1. ^{1}H-NMR spectra in $CDCl_3$, (300 MHz) of polymer **HP1**

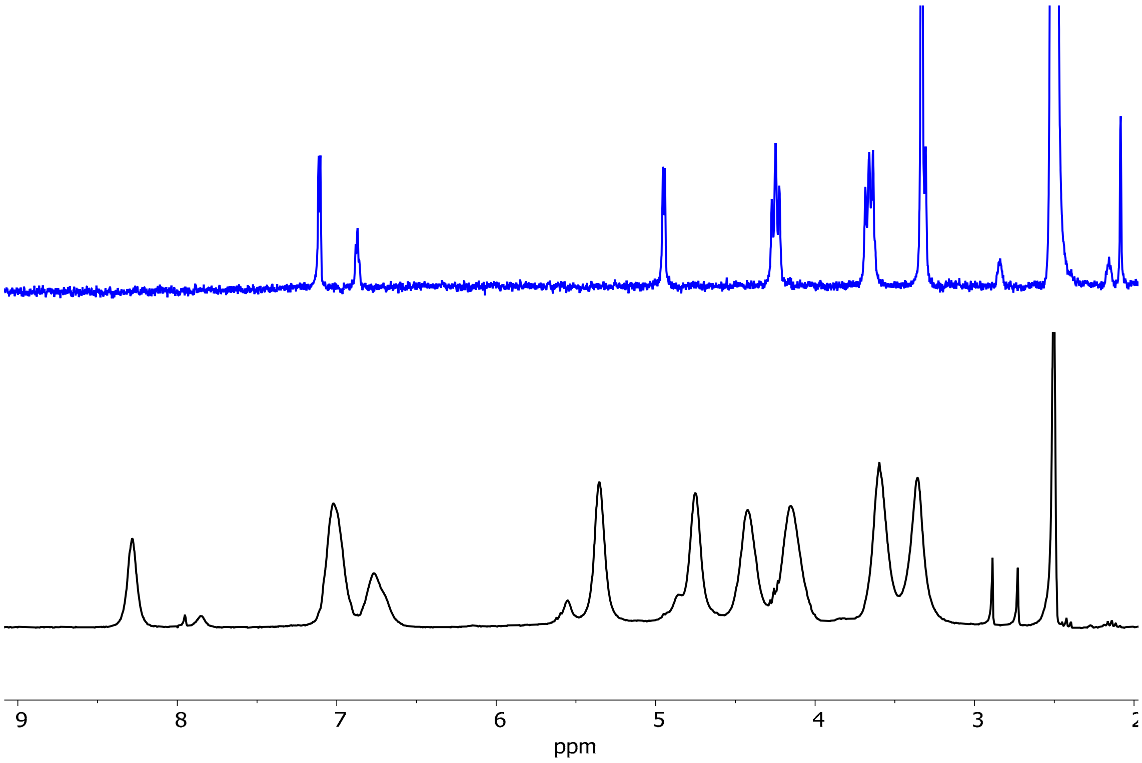

Figure S2. Stacked ^{1}H NMR of compound **4** (blue line) and polymer **HP1** (black line) in DMSO-d_6 (300 MHz).

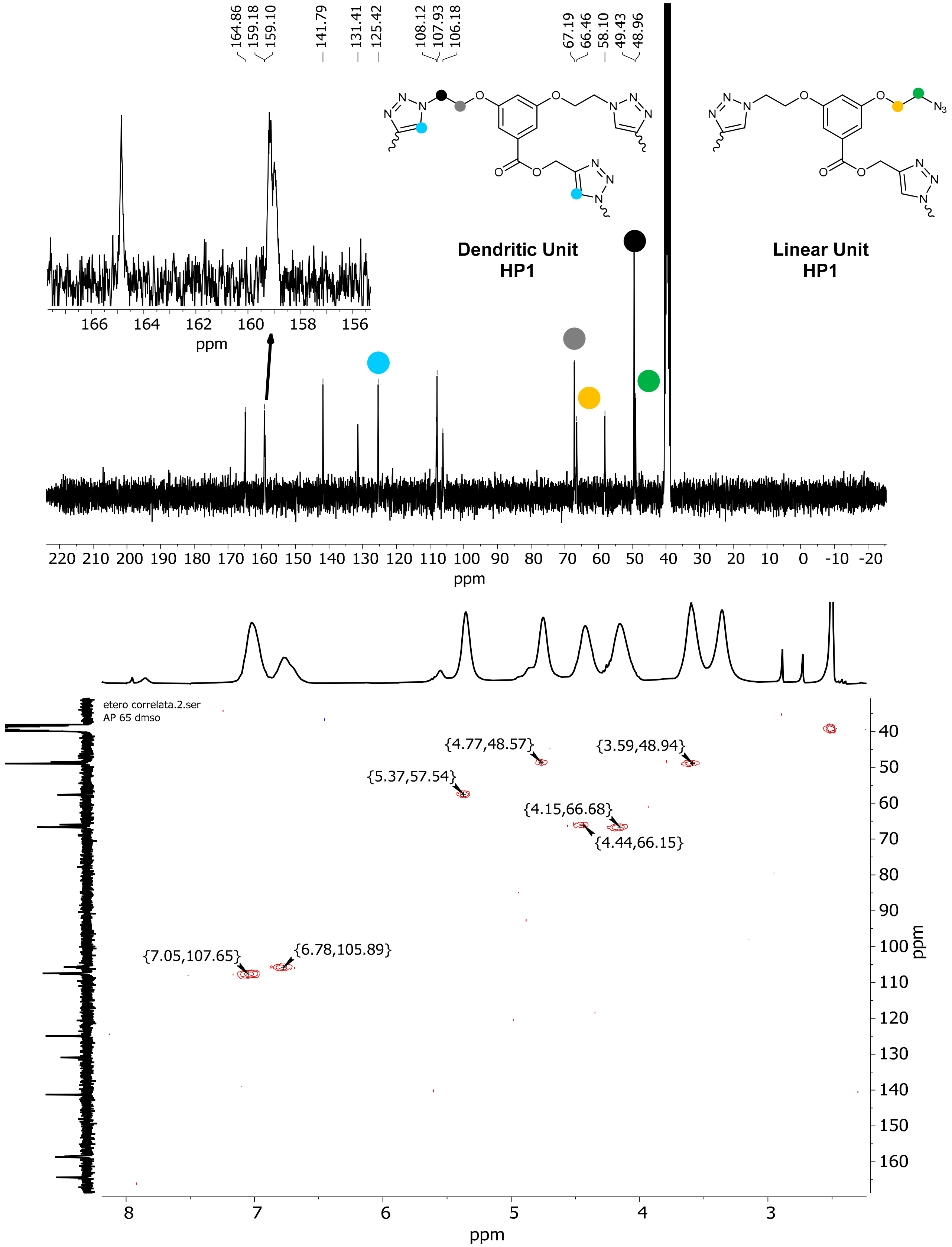

Figure S3. ^{13}C-NMR spectra (top) and HSQC spectra (bottom) in $CDCl_3$ of polymer **HP1**. Signals at 48.96 and 66.46 ppm were attributed to the **C**H_2 carbons of linear units, while signals at 49.43 and 67.19 ppm were attributed to the **C**H_2 carbons of dendritic units. Signal at 125.4 ppm was assigned to the triazole C–H carbon.

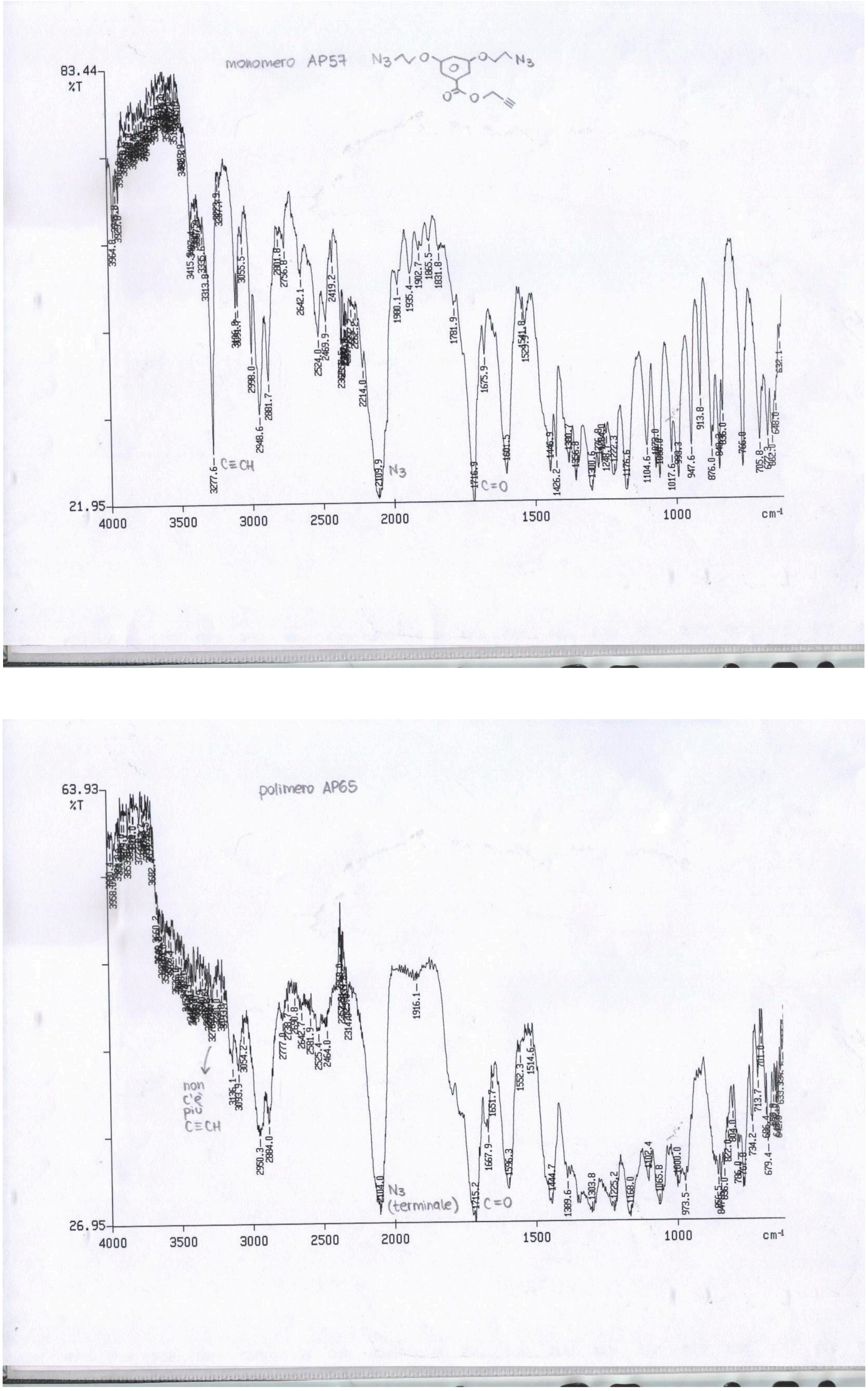

Figure S4. FTIR spectra of compound **3** (Top) and polymer **HP1** (Bottom) respectively. In the spectra of polymer **HP1** the triple bond CH stretching at 3277 cm^{-1} is absent.

3. Characterization of HP2

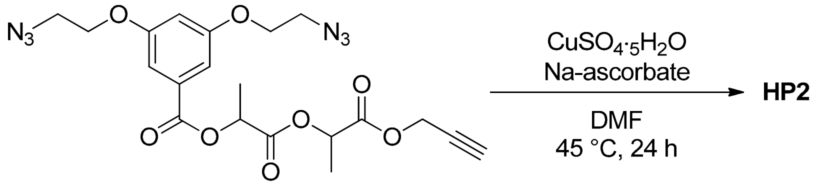

9a

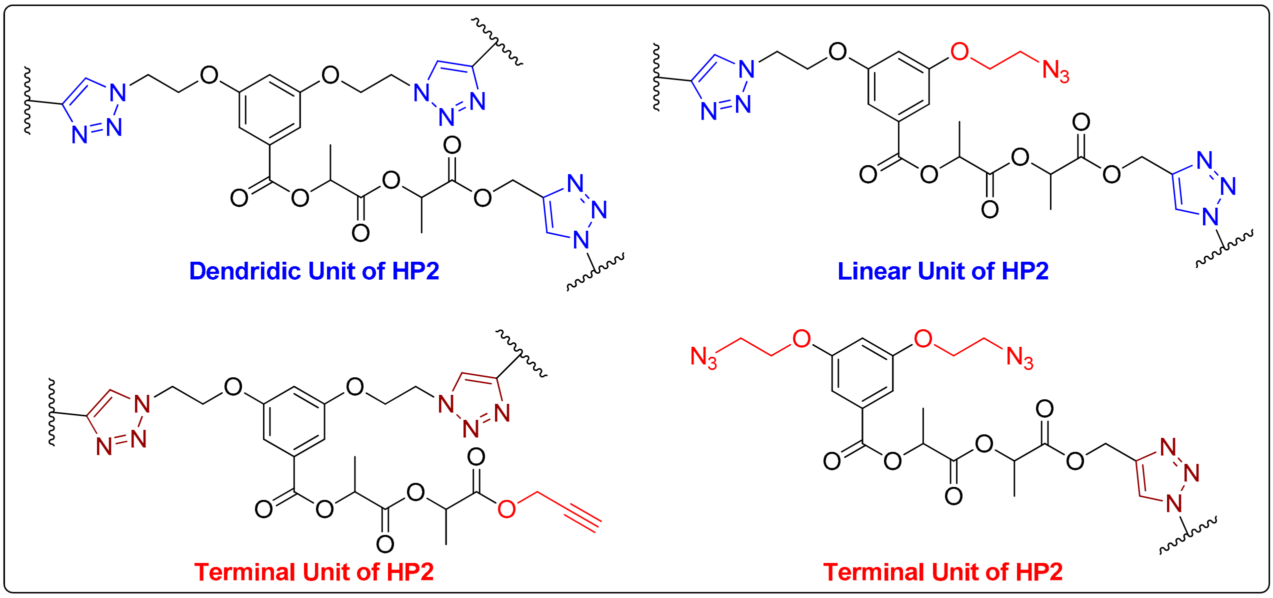

In Figure S5 and S6 we report the ^{1}H NMR spectra of polymer **HP2** and the stacked ^{1}H NMR spactra of **HP2** with compound **9a**, while in Figure S7 we report the ^{13}C NMR and HSQC spectra of **HP2** in DMSO-d_6.

By using the relative integration in the ^{1}H NMR spectra of Figure S5 we obtained L = 2.24 (from peak at 4.17 univocal for L), and D = (2.17*2/3, from peak 4.76 in which both D and L proton resonances, the former doubling the latetr in terms of number of protons, are present and superimposed) = 1.44. Using this value of L and D we obtain a DB = 0.39

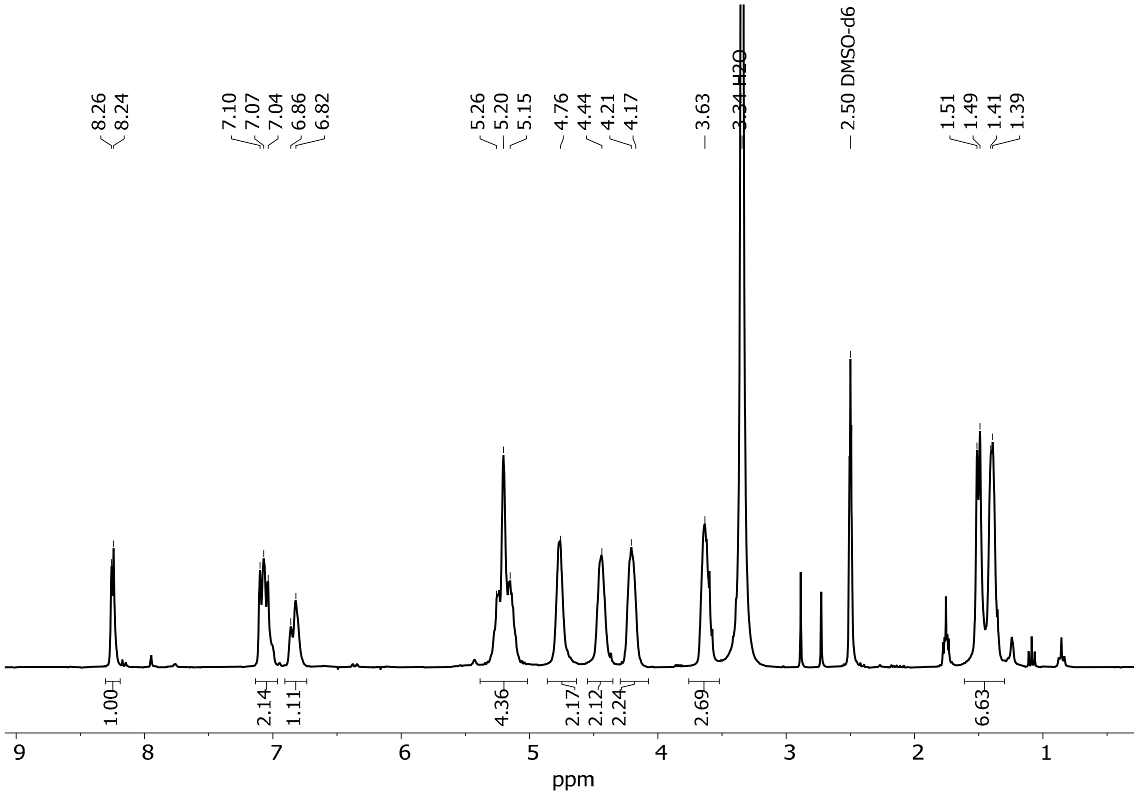

Figure S5. ^{1}H-NMR spectra in $CDCl_3$, (300 MHz) of polymer **HP2**

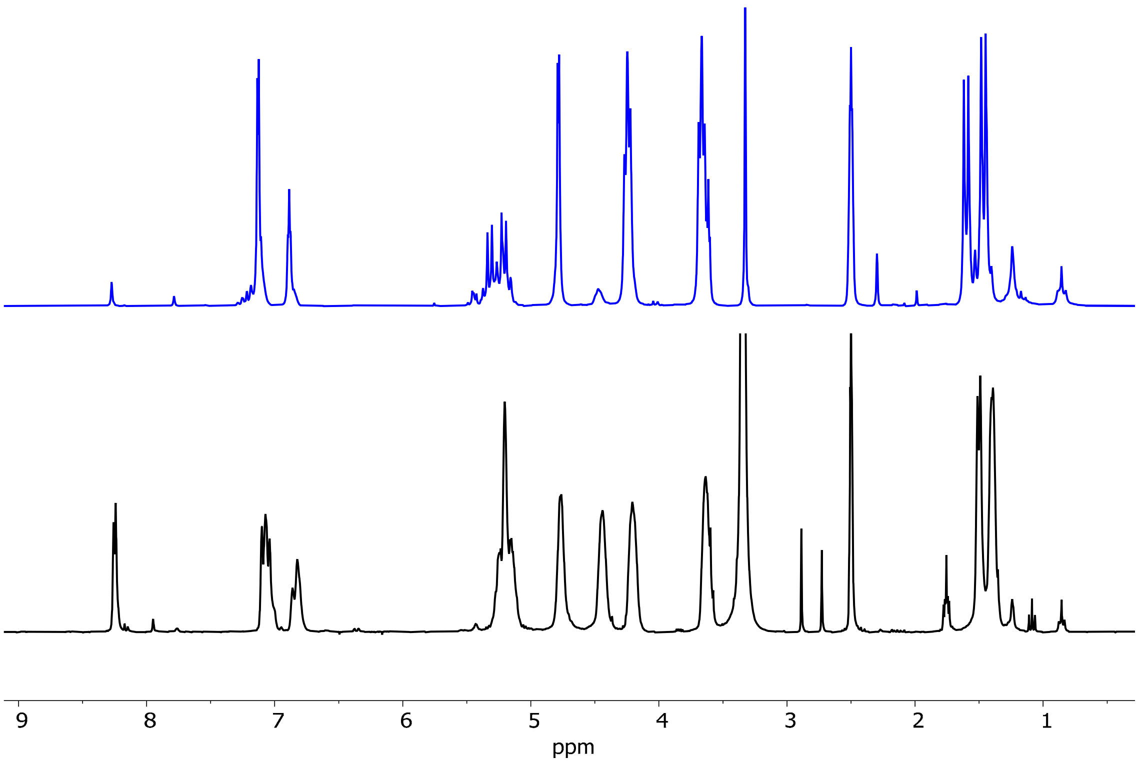

Figure S6. Stacked ^{1}H NMR of compound **9a** (blue line) and polymer **HP2** (black line) in DMSO-d_6 (300 MHz).

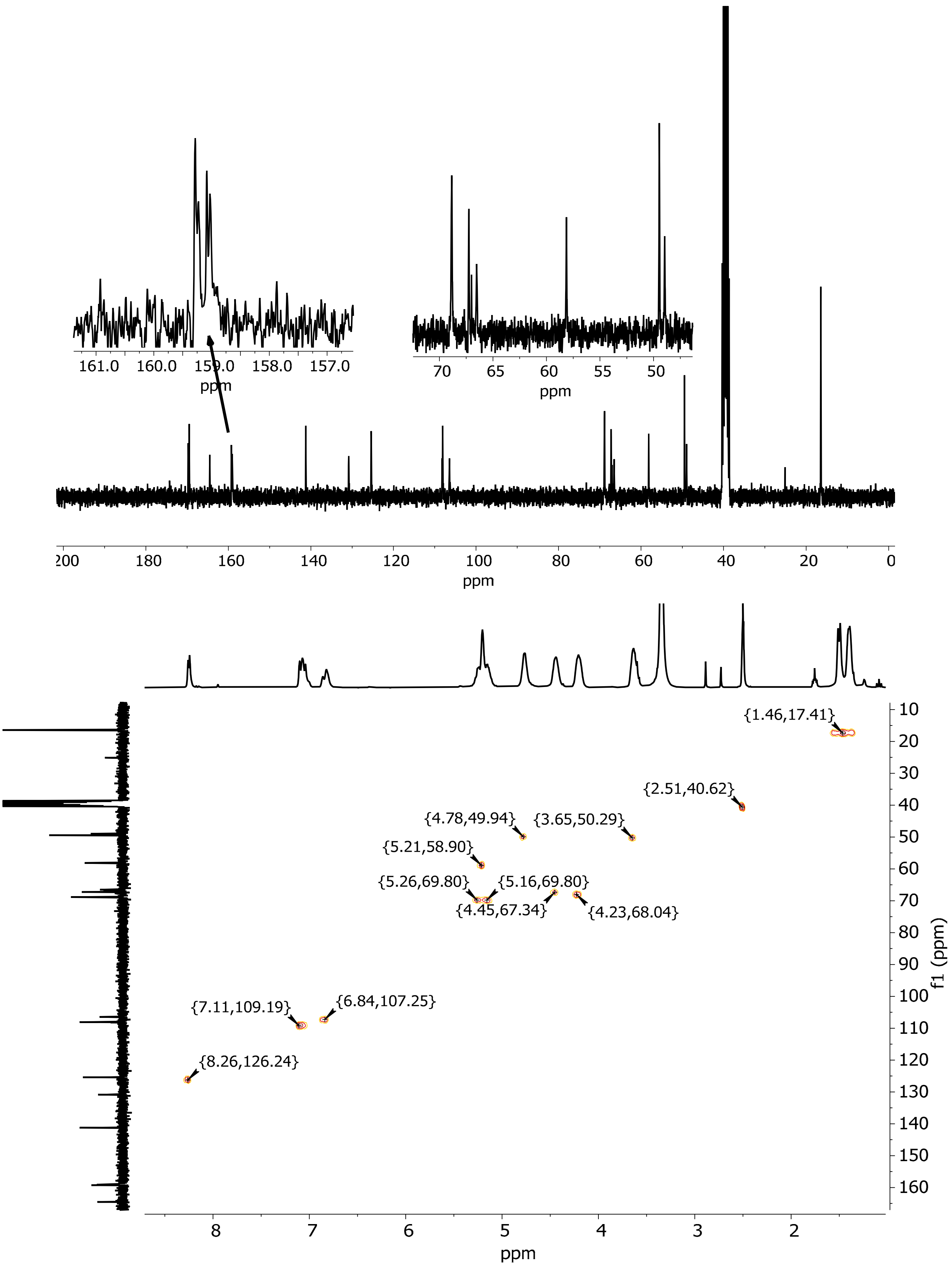

Figure S7. ^{13}C-NMR spectra (top) and HSQC spectra (bottom) in $CDCl_3$ of polymer **HP2**.

4. Characterization of HP3

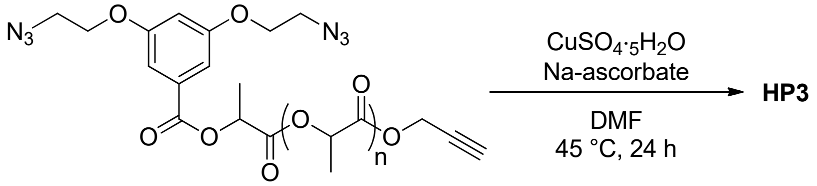

9b n=3

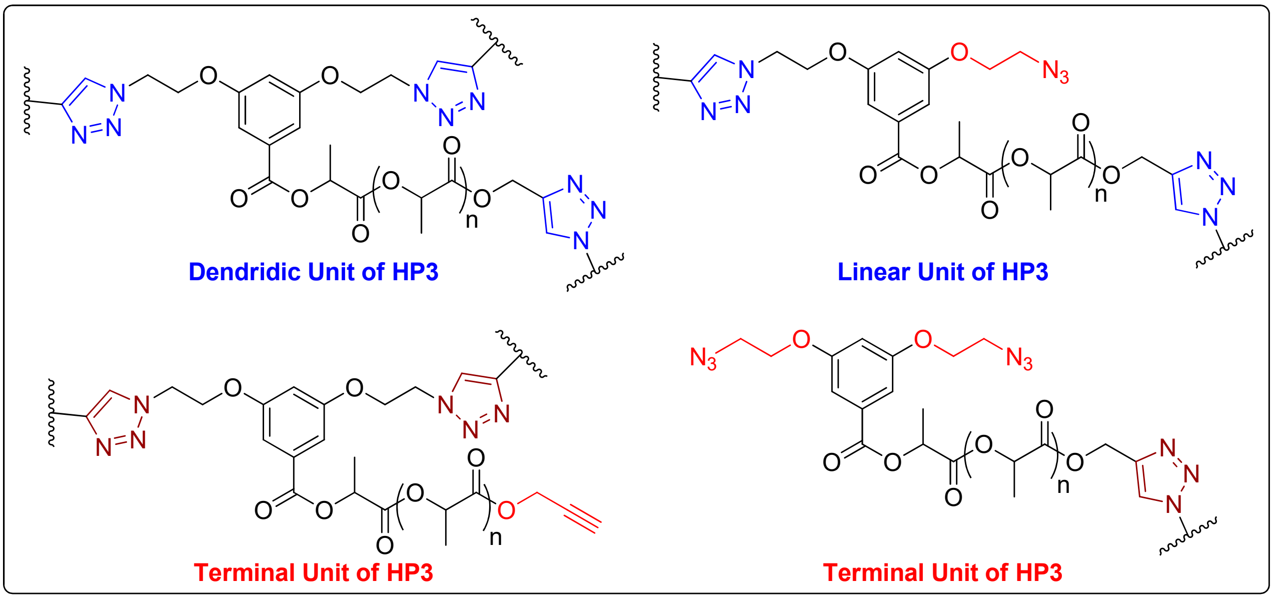

In Figure S8 and S9 we report the ^{1}H NMR spectra of polymer **HP3** and the stacked ^{1}H NMR spectra of **HP3** with compound **9b**, while in Figure S10 we report the ^{13}C NMR and HSQC spectra of **HP3** in DMSO-d_6. By using the relative integration in the ^{1}H NMR spectra of Figure S5 we obtained L = 2.55 (from peak at 4.22 ppm univocal for L), and D = (1.89*2/3, from peak 4.74 ppm in which both D and L proton resonances, the former doubling the latter in terms of number of protons, are present and superimposed) = 1.26. Using this value of L and D we obtain a DB = 0.33

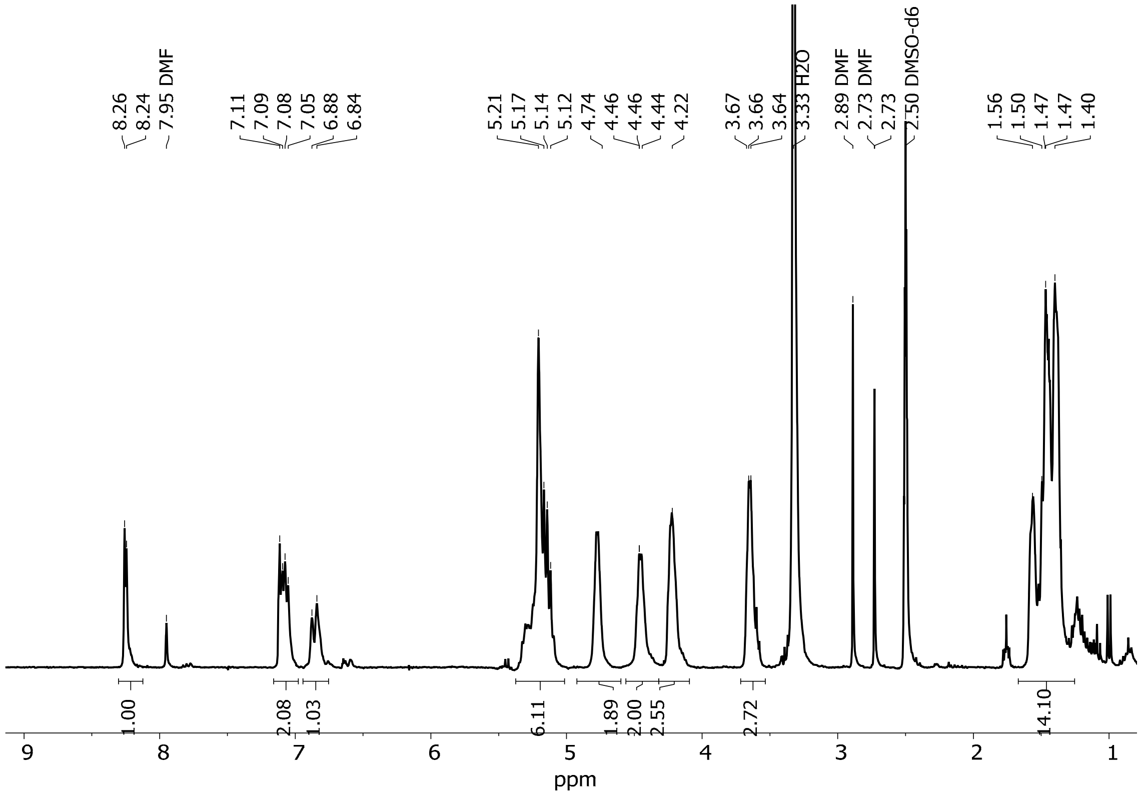

Figure S8. ^{1}H-NMR spectra in $CDCl_3$, (300 MHz) of polymer **HP3**

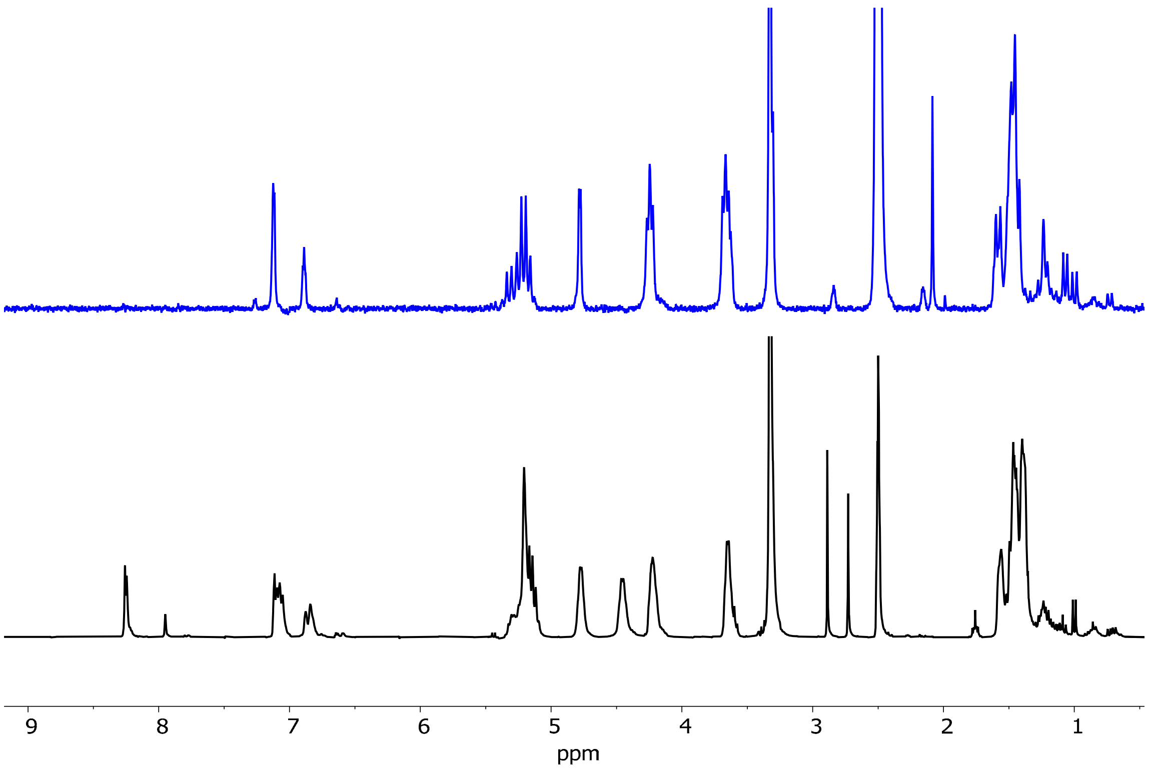

Figure S9. Stacked ^{1}H NMR of compound **9c** (blue line) and polymer **HP3** (black line) in DMSO-d_6 (300 MHz).

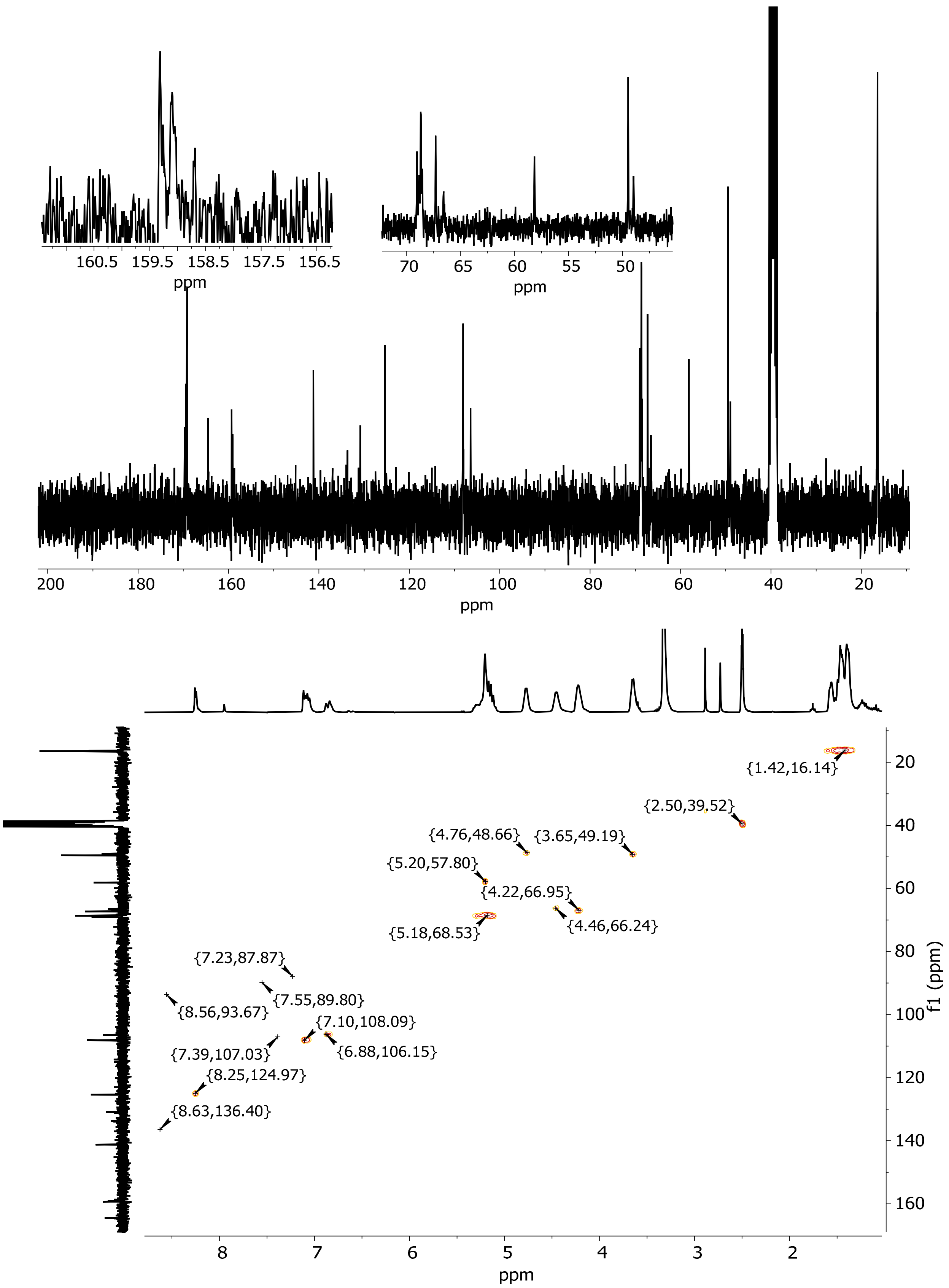

Figure S10. ^{13}C-NMR spectra (top) and HSQC spectra (bottom) in $CDCl_3$ of polymer **HP4**.

5. Thin Film Experiments

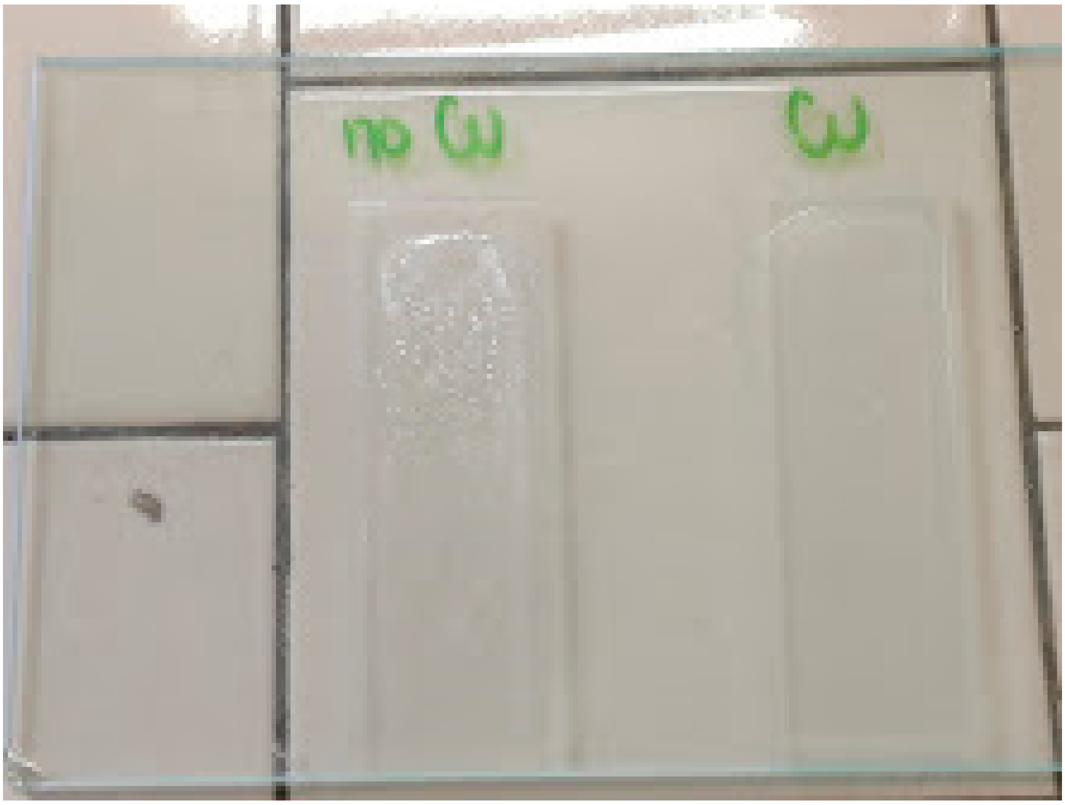

Figure S11. Thin film deposition by drop casting. Left: solution containing monomer **4** (10.3 mg, $3.12*10^{-5}$ mol) and ascorbic acid (1 mg, $5.24 *10^{-6}$ mol) in DMF (100 µL). Right: solution containing monomer **4** (10.3 mg, $3.12*10^{-5}$ mol), ascorbic acid (1 mg, $5.24 *10^{-6}$ mol) and $CuSO_4 \cdot 5H_2O$ (0.257 mg, $1.03*10^{-6}$ mol) in DMF (100 µL). Pictures taken after 16 h from deposition, at room temperature.

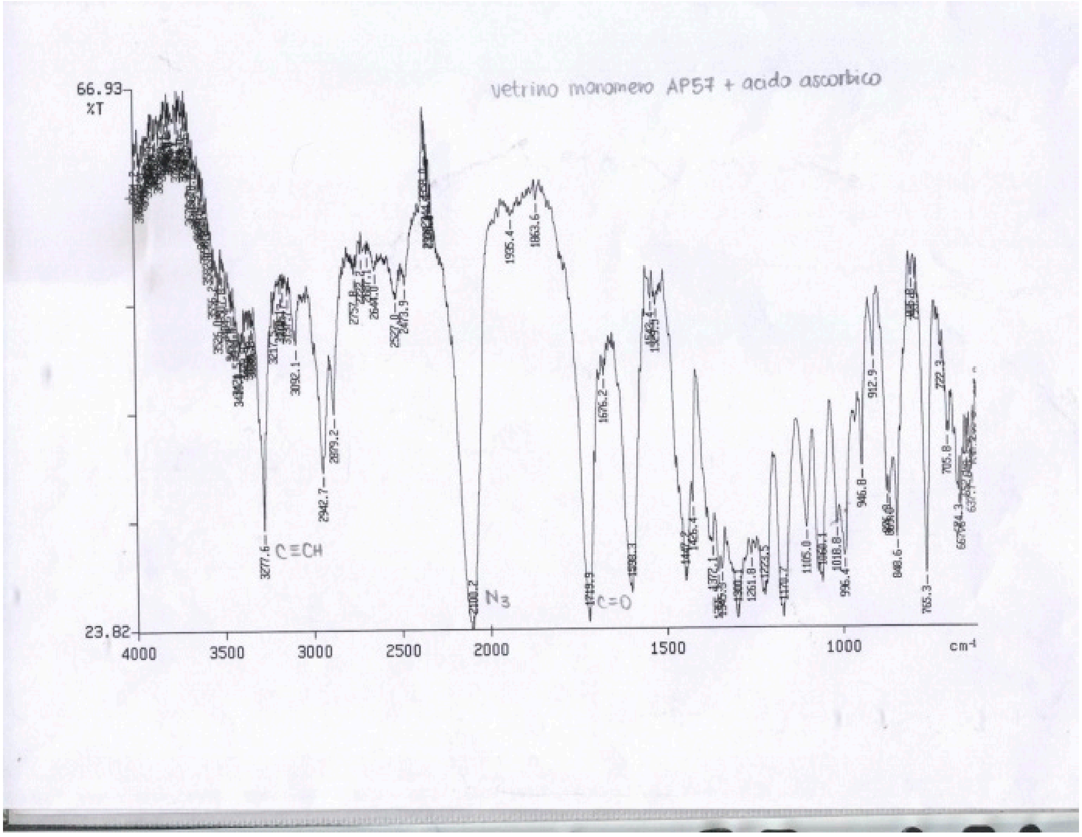

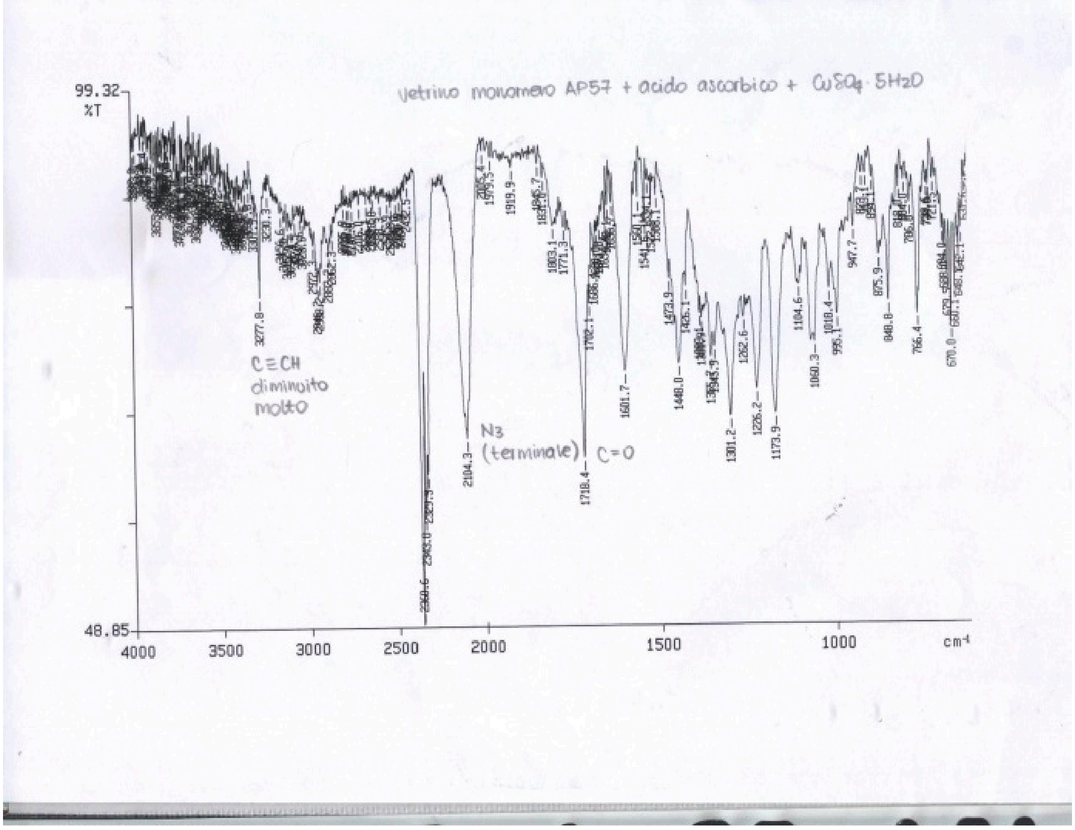

Figure S12. FTIR characterization of the material deposited, taken after 16 h from deposition, at room temperature. Top: control, slide on the left in Figure S11. Bottom: reaction, slide on the right in Figure S11

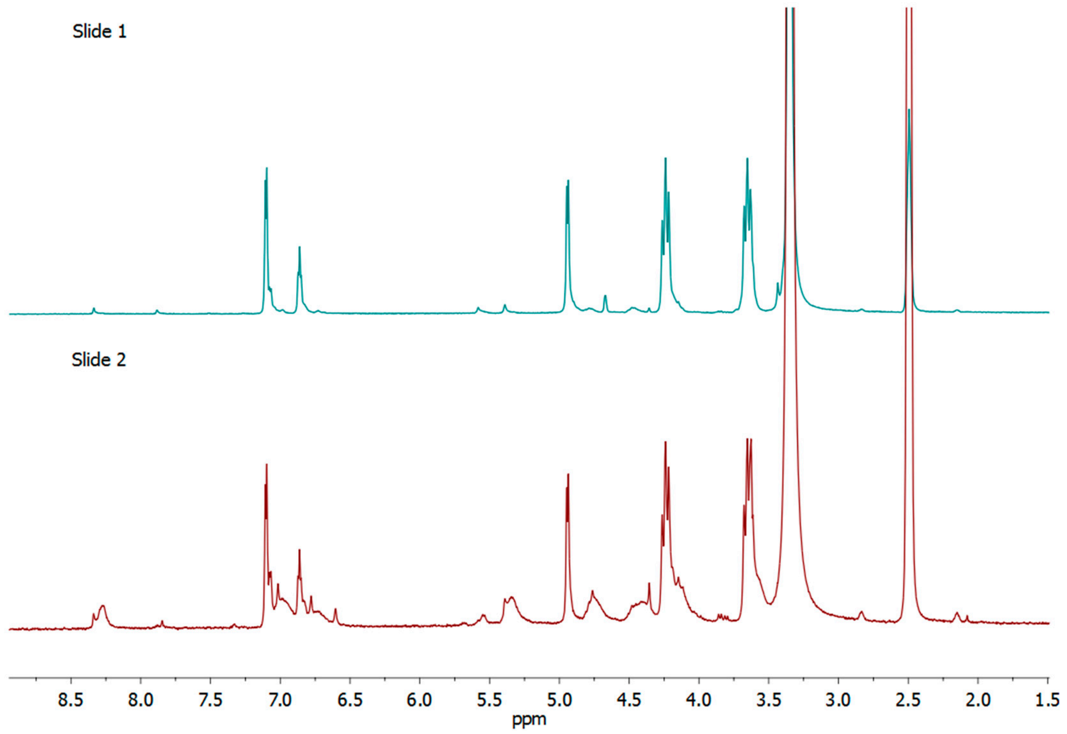

Figure S13. ^{1}H NMR characterization of the material deposited, taken after 16 h from deposition, at room temperature. Top: control, slide on the left in Figure S11. Bottom: reaction, slide on the right in Figure S11.

6. Characterization of New Compounds

Compound 2

^{1}H NMR (DMSO-d_6, 300 MHz)

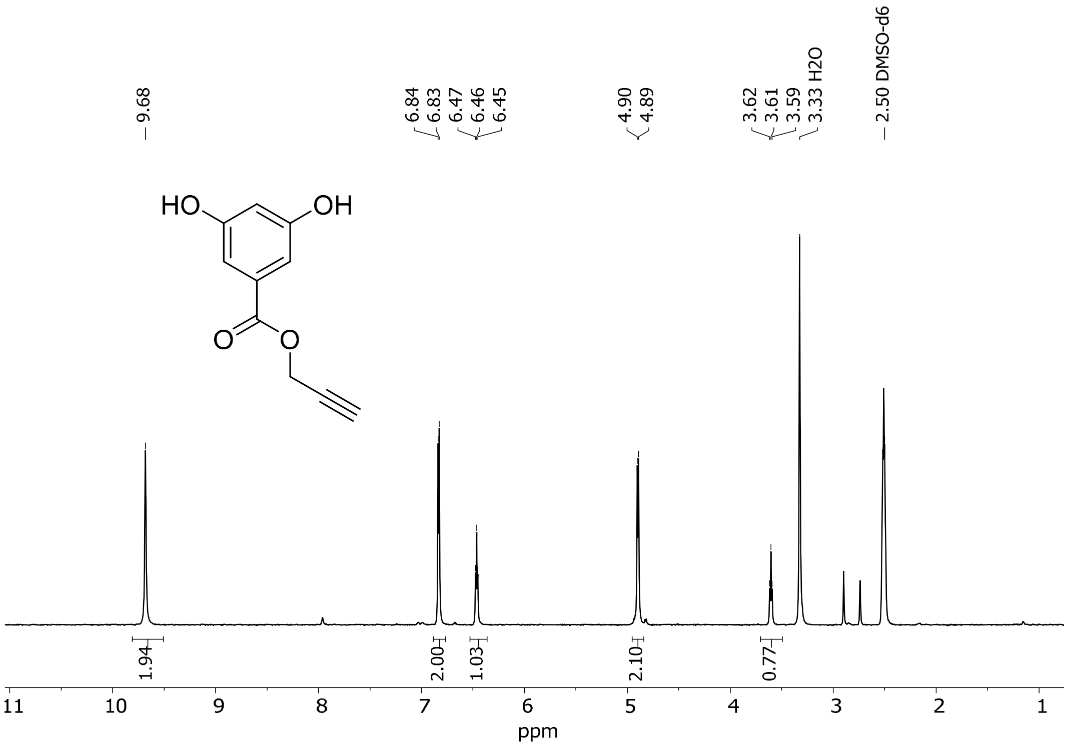

^{13}C-NMR (DMSO-d6, 300 MHz) spectrum

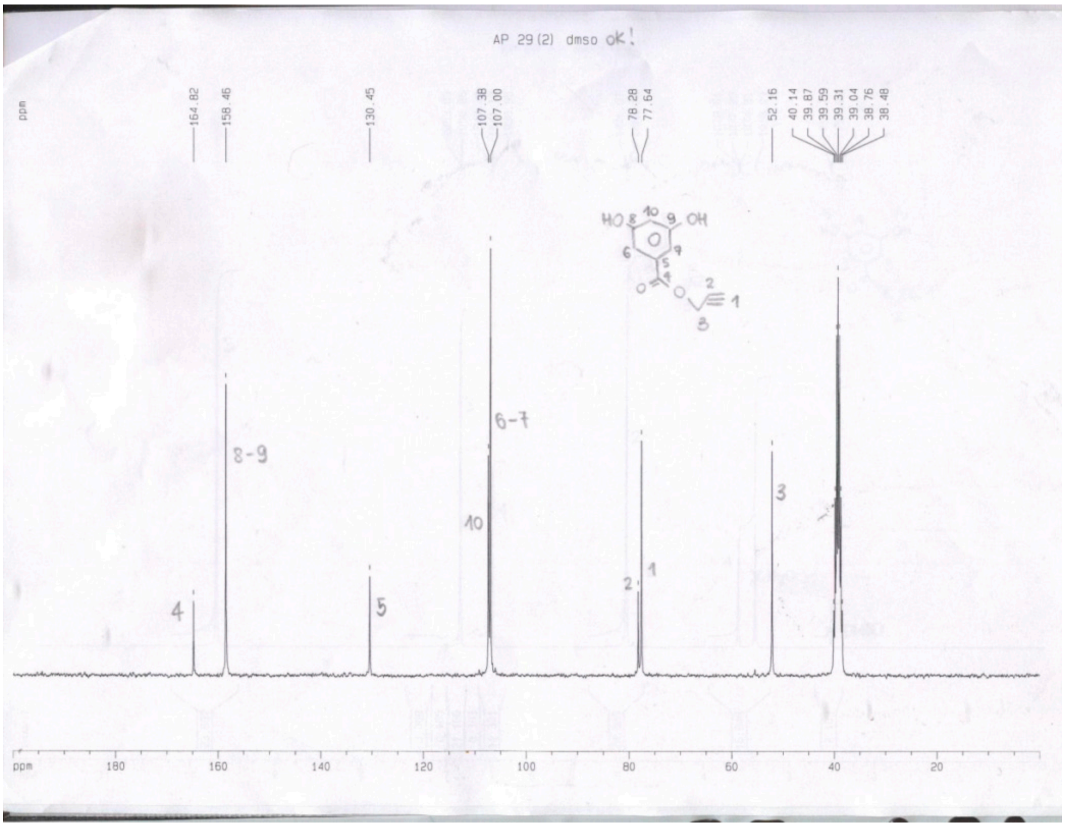

ESI-MS

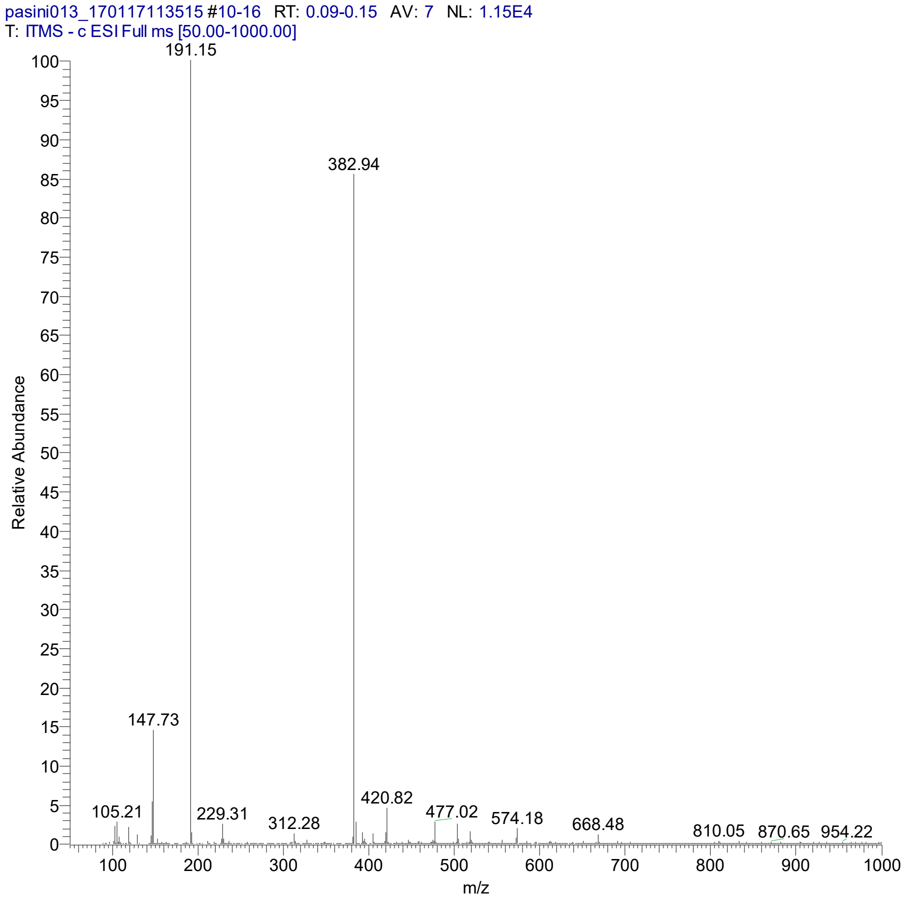

pasini013_170117113515 #10-16 RT: 0.09-0.15 AV: 7 NL: 1.15E4
T: ITMS - c ESI Full ms [50.00-1000.00]
Relative Abundance
m/z
191.15
382.94
147.73
105.21
229.31
312.28
420.82
477.02
574.18
668.48
810.05
870.65
954.22

Compound S2

^{1}H-NMR (CDCl$_3$, 300 MHz)

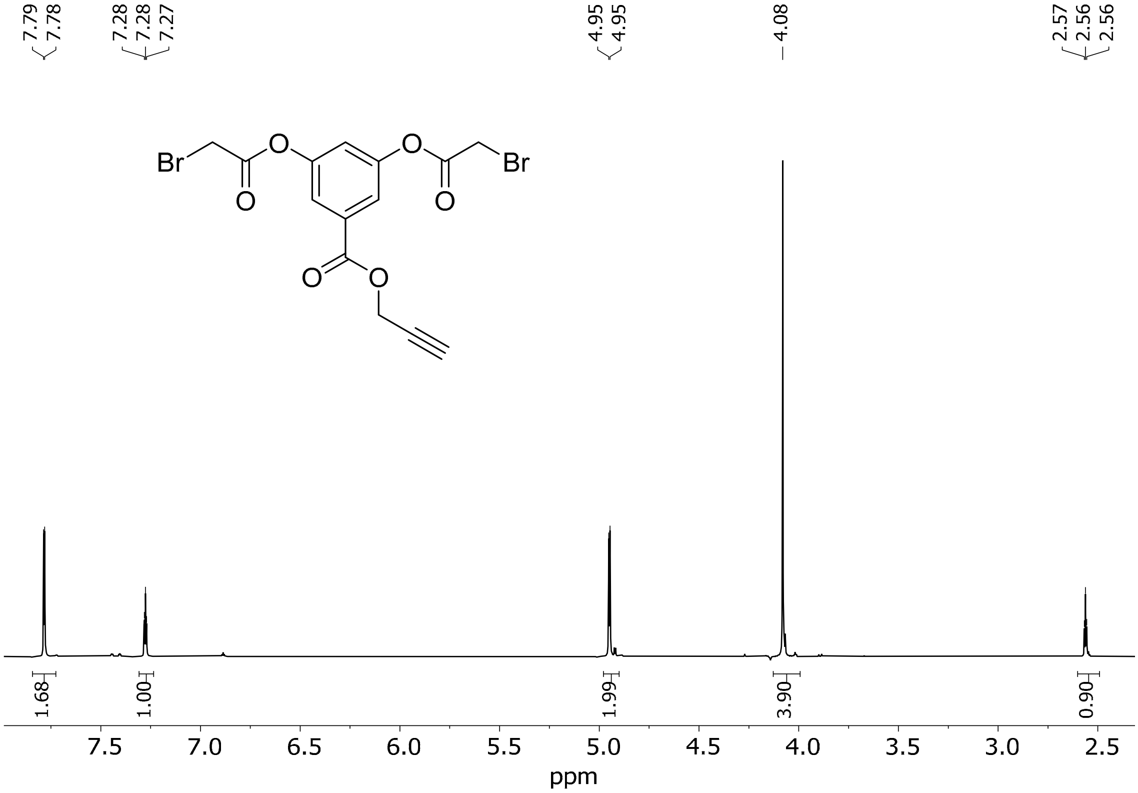

^{13}C-NMR (CDCl$_3$, 300 MHz)

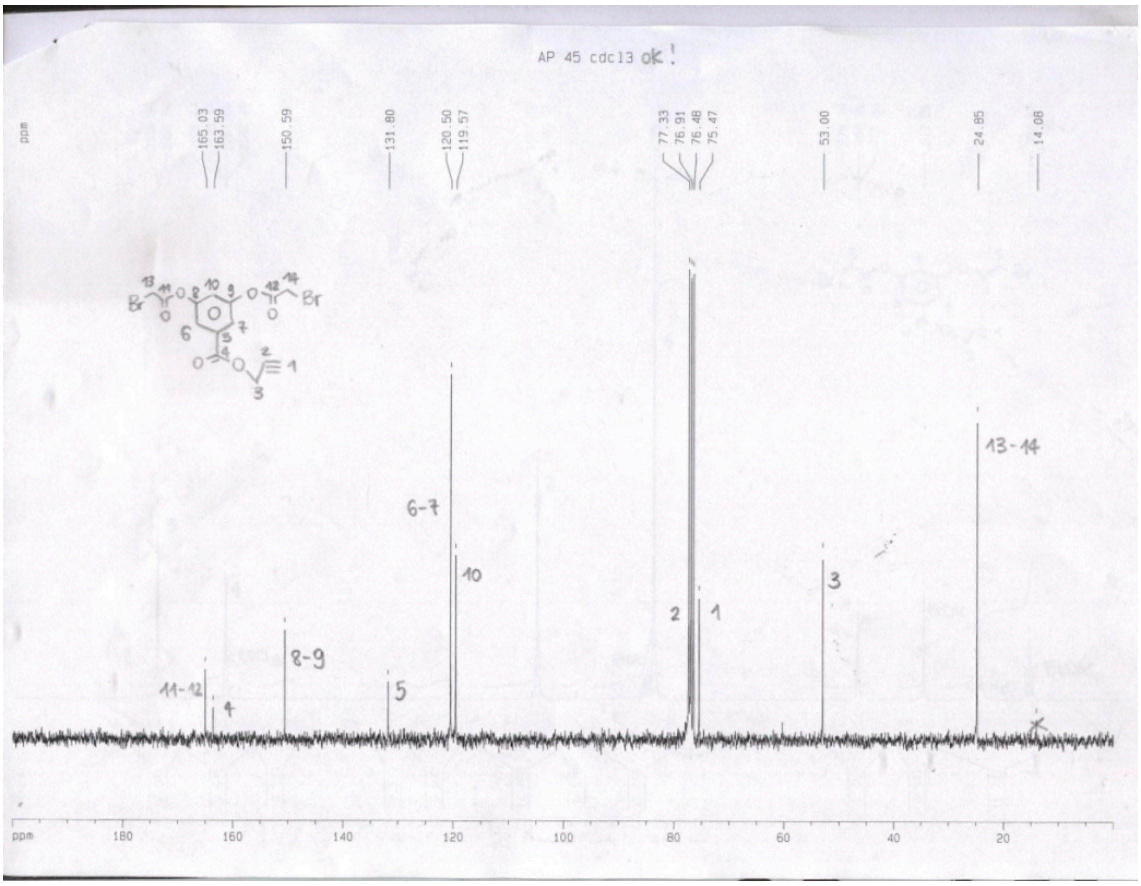

ESI-MS

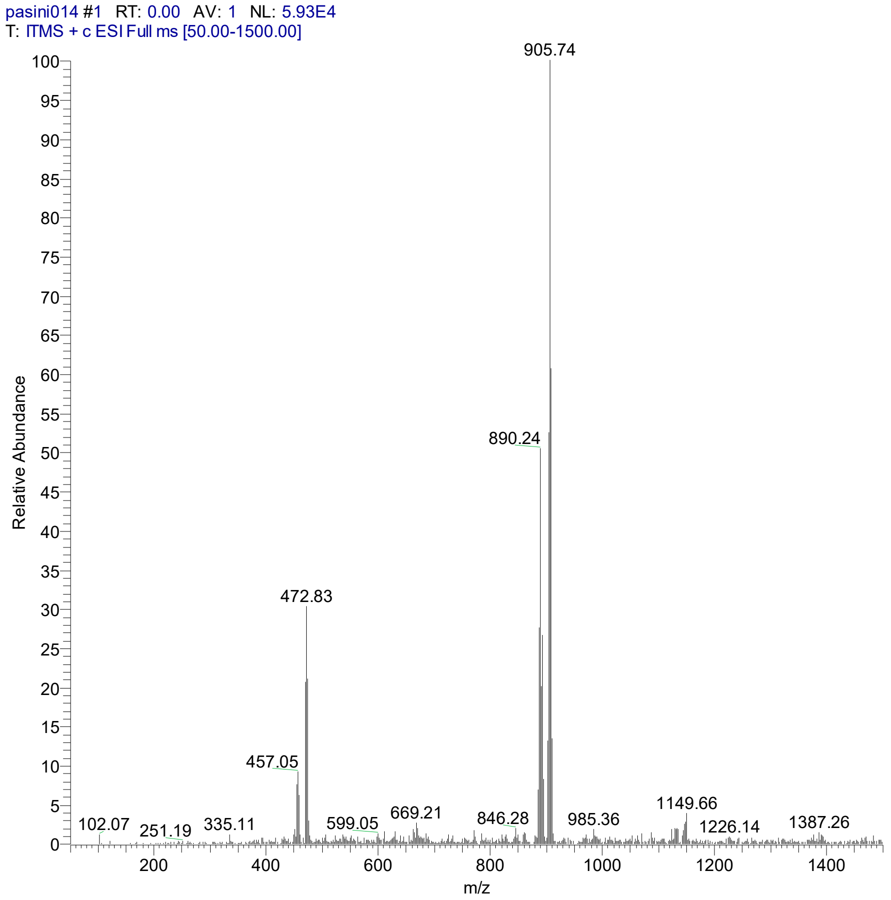
pasini014 #1 RT: 0.00 AV: 1 NL: 5.93E4
T: ITMS + c ESI Full ms [50.00-1500.00]
905.74
890.24
472.83
457.05
102.07
251.19
335.11
599.05
669.21
846.28
985.36
1149.66
1226.14
1387.26
Relative Abundance
m/z

Compound 3

^{1}H-NMR ($CDCl_3$, 300 MHz)

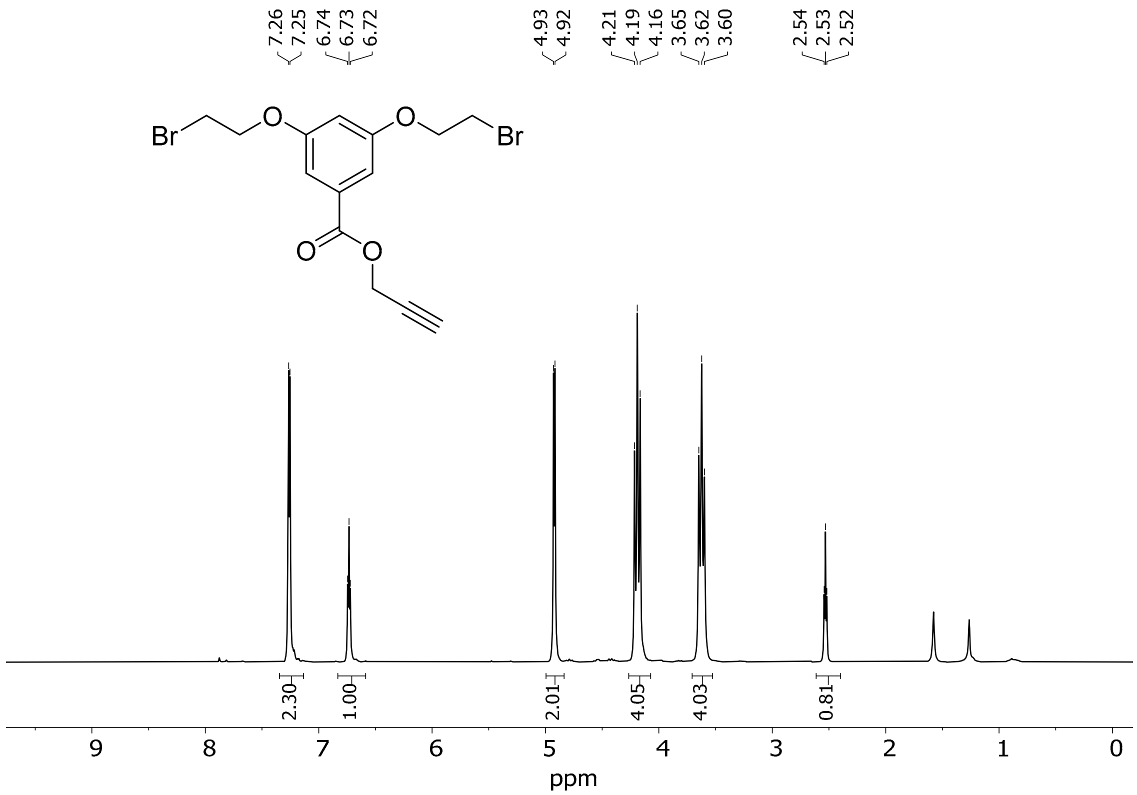

^{13}C-NMR ($CDCl_3$, 300 MHz)

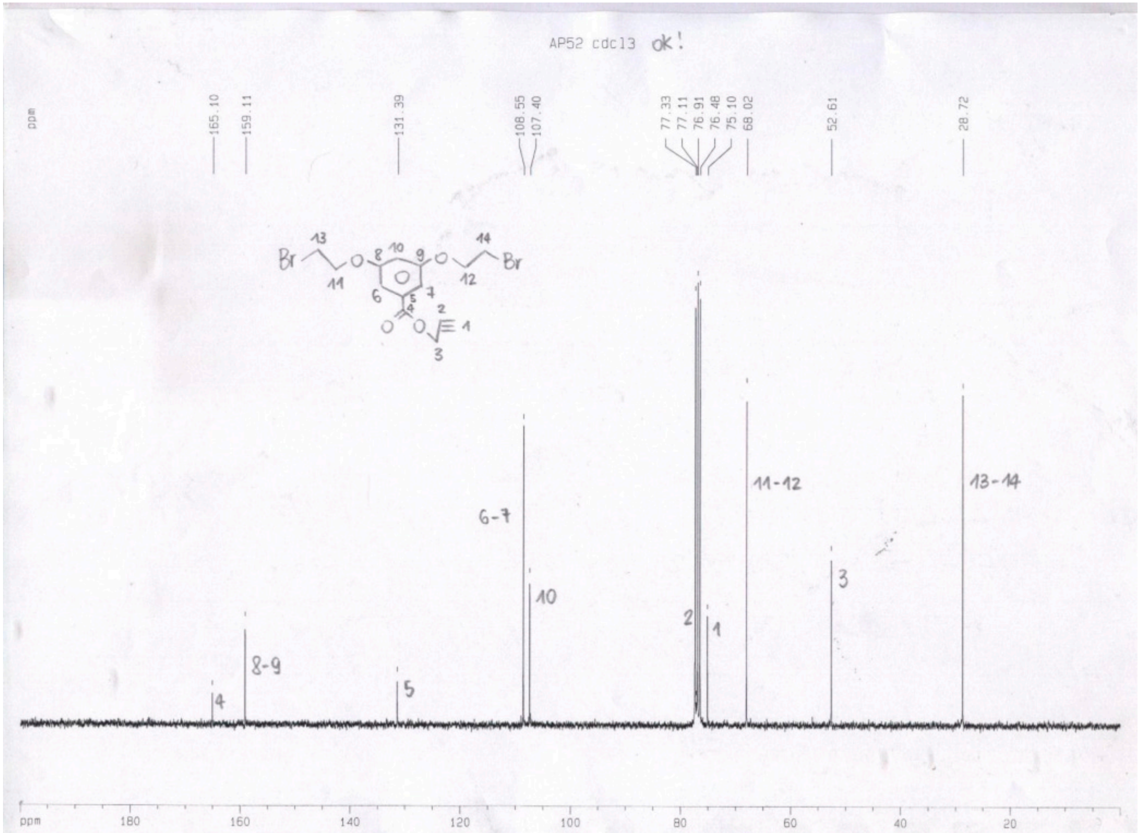

ESI-MS

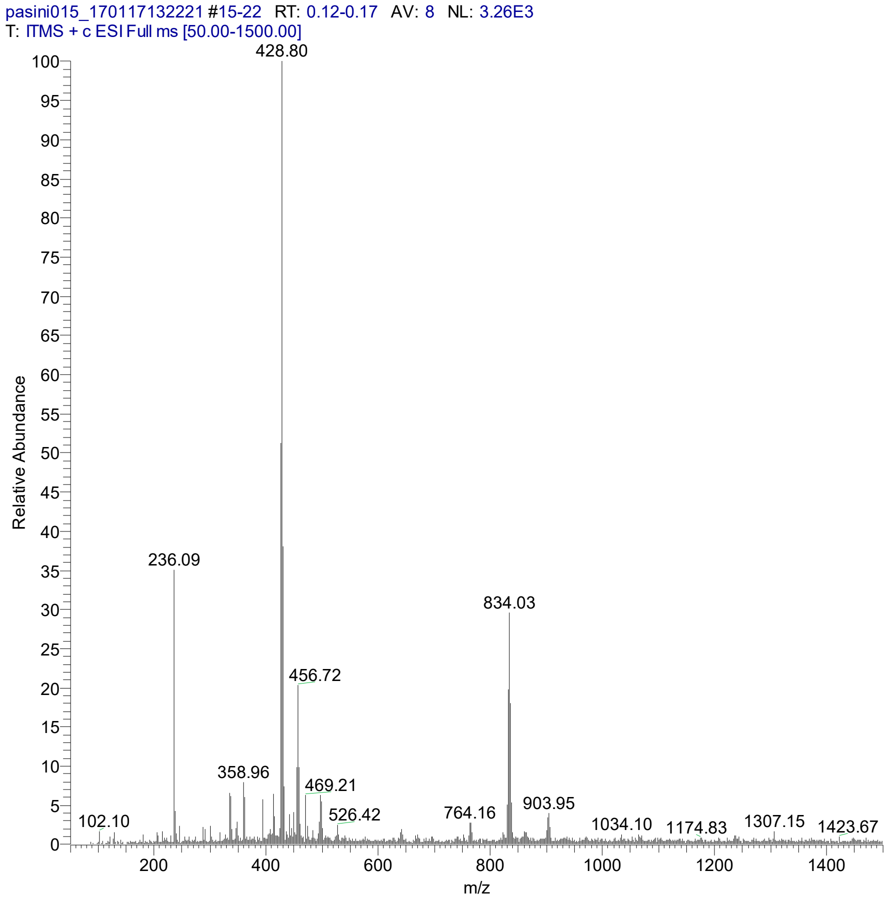

pasini015_170117132221 #15-22 RT: 0.12-0.17 AV: 8 NL: 3.26E3
T: ITMS + c ESI Full ms [50.00-1500.00]
Relative Abundance
m/z
428.80
236.09
834.03
456.72
358.96
469.21
526.42
764.16
903.95
102.10
1034.10
1174.83
1307.15
1423.67

Compound 4

^{1}H-NMR (CDCl$_3$, 200 MHz)

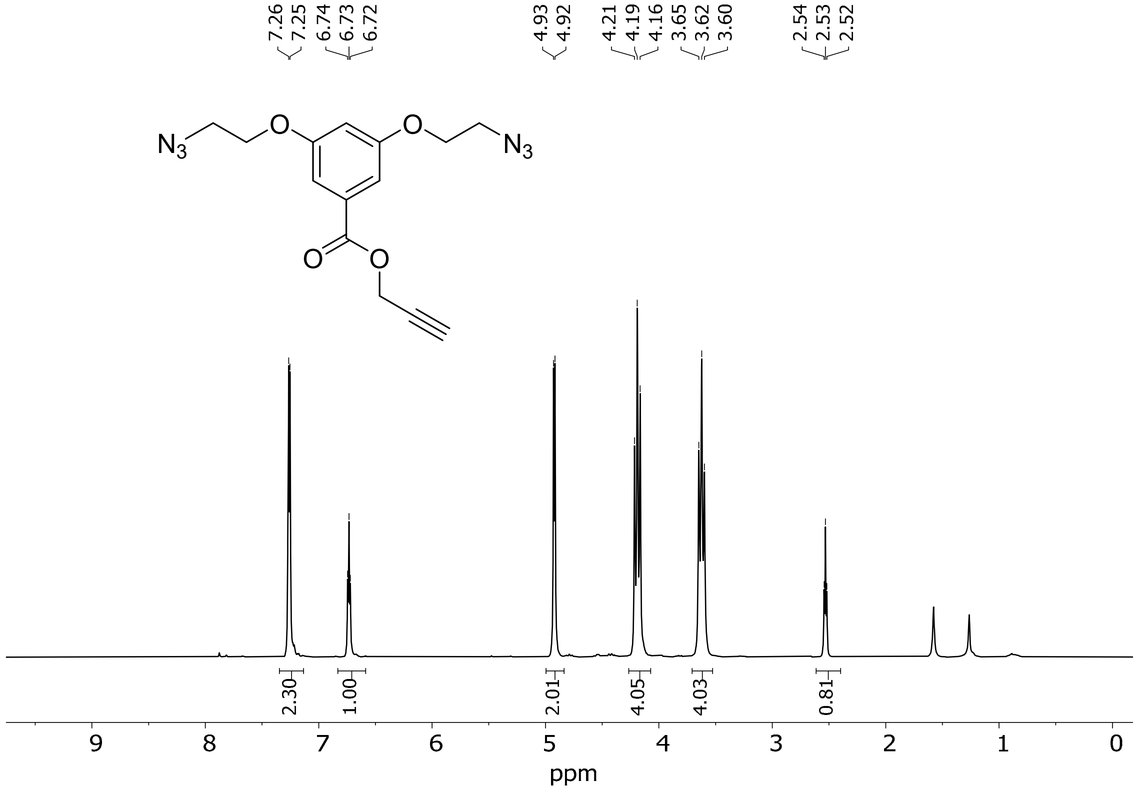

^{13}C-NMR (CDCl$_3$, 300 MHz)

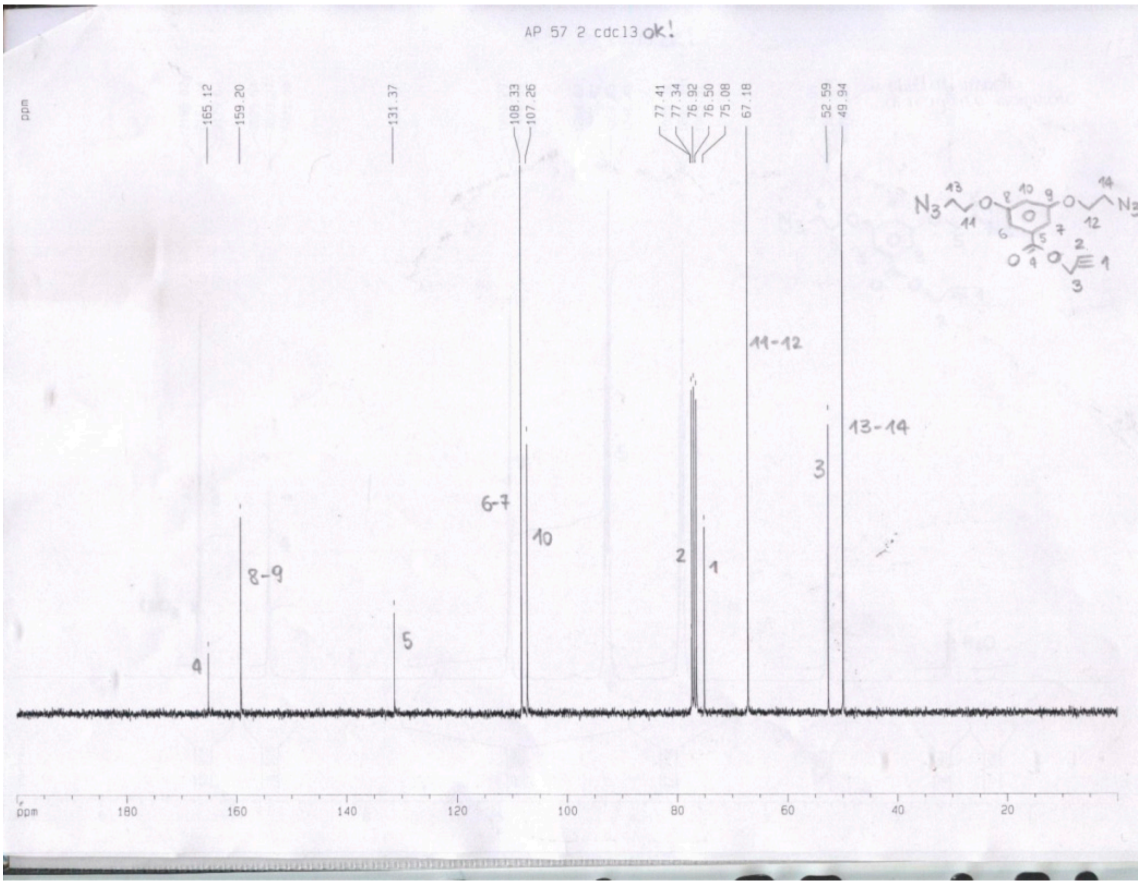

ESI-MS

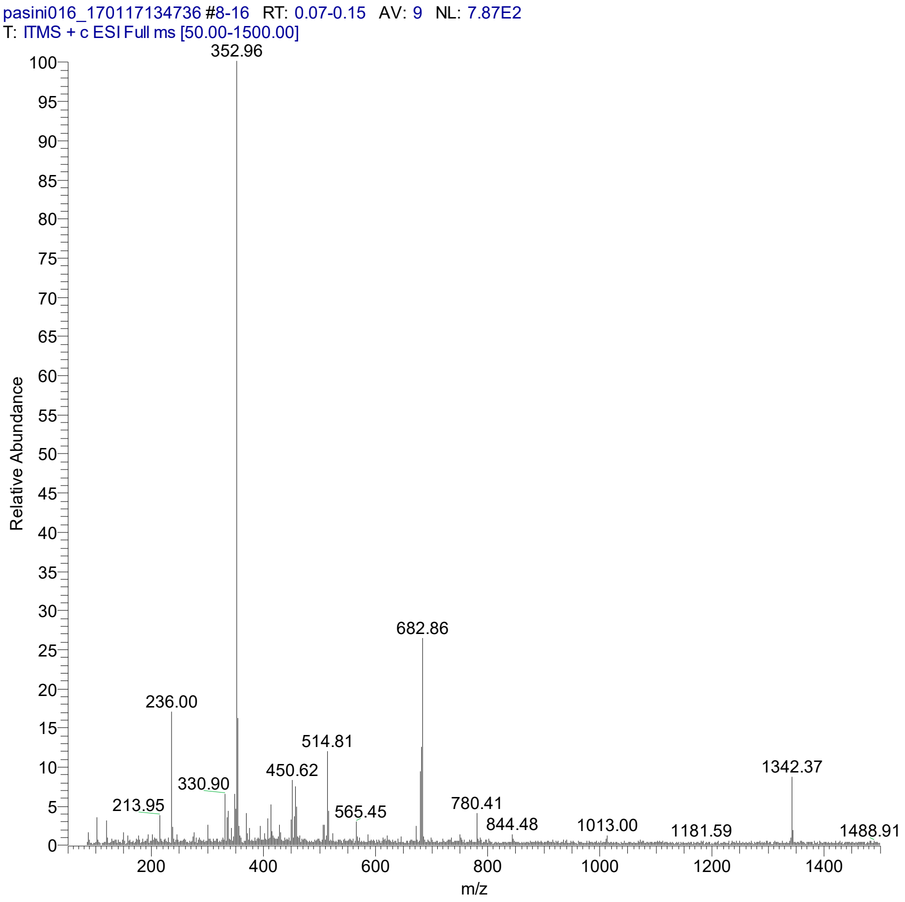

pasini016_170117134736 #8-16 RT: 0.07-0.15 AV: 9 NL: 7.87E2
T: ITMS + c ESI Full ms [50.00-1500.00]
352.96
682.86
236.00
514.81
450.62
330.90
213.95
565.45
780.41
844.48
1013.00
1181.59
1342.37
1488.91
Relative Abundance
m/z

Compound 9a

^{1}H-NMR (CDCl$_3$, 200 MHz) spectrum.

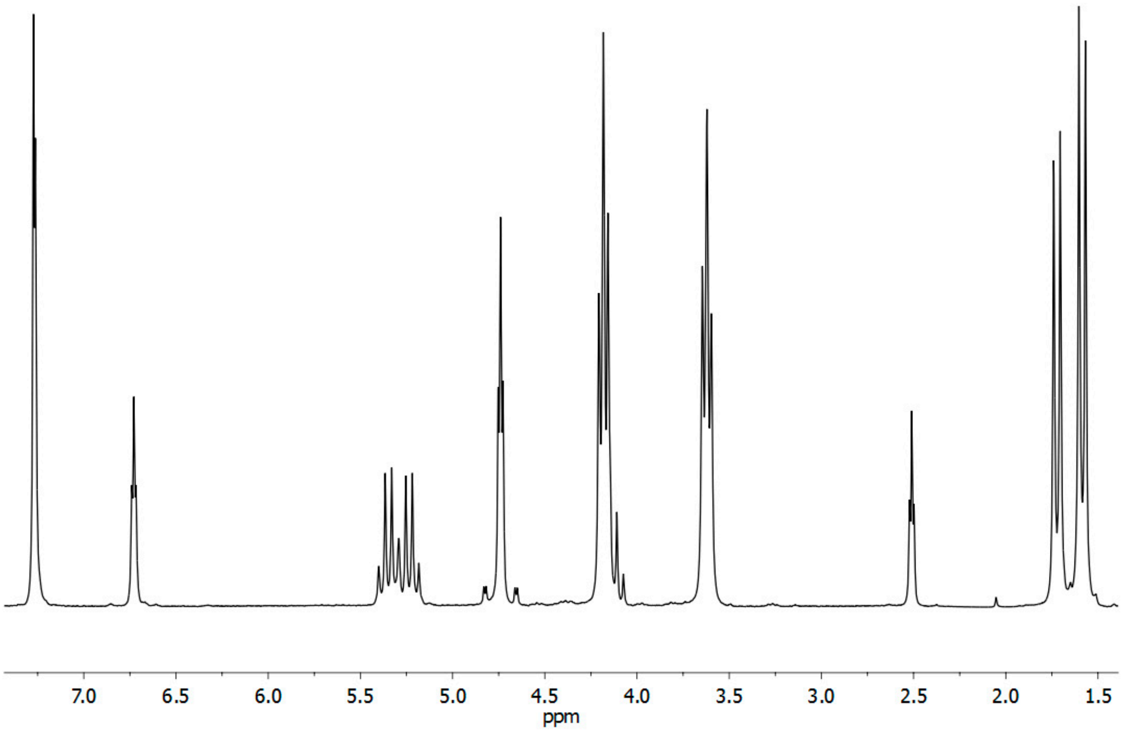

^{13}C-NMR (CDCl$_3$, 300 MHz)

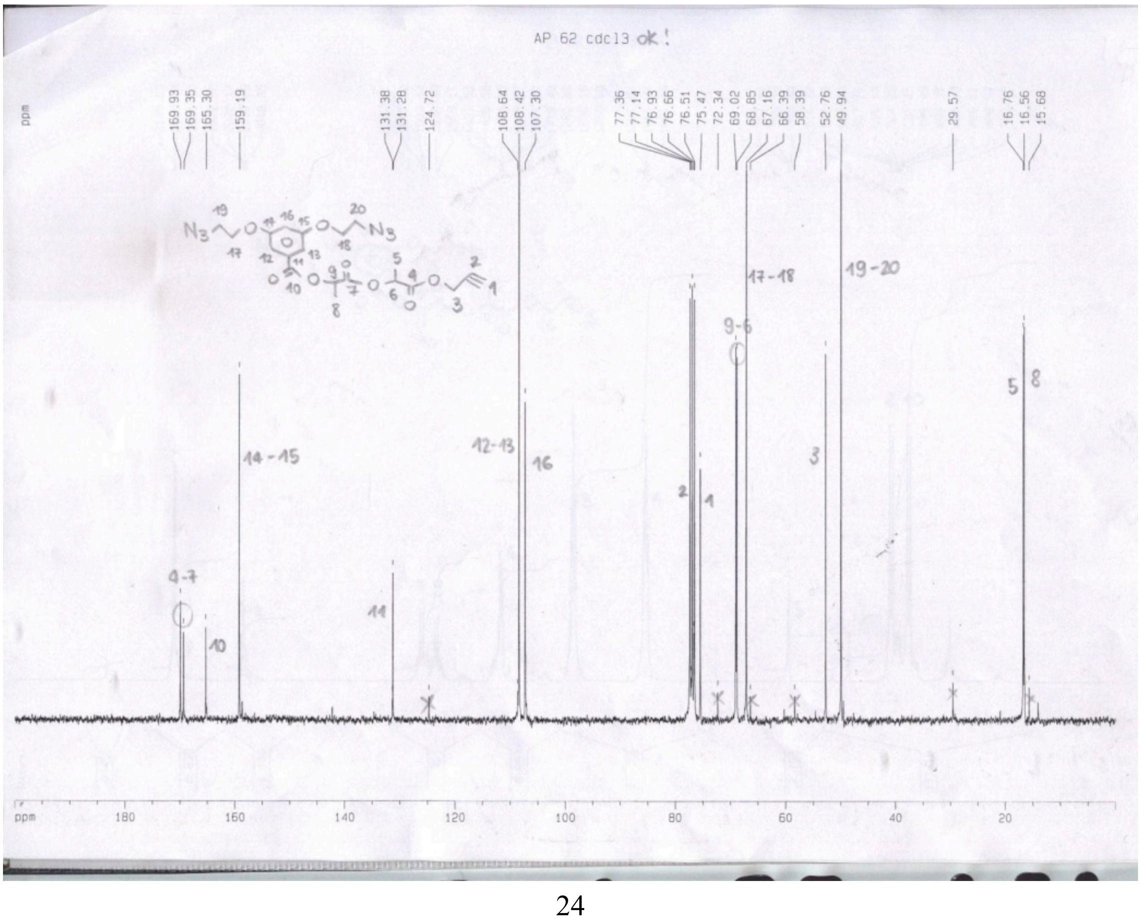

ESI-MS

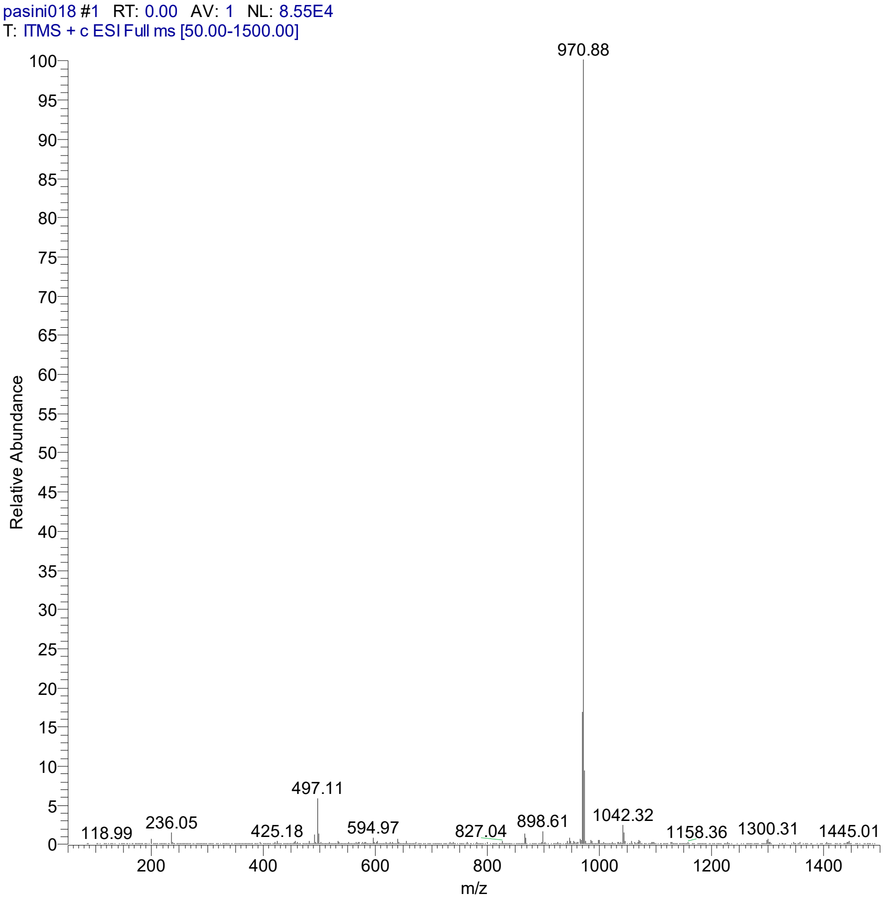

pasini018 #1 RT: 0.00 AV: 1 NL: 8.55E4
T: ITMS + c ESI Full ms [50.00-1500.00]
970.88
497.11
118.99
236.05
425.18
594.97
827.04
898.61
1042.32
1158.36
1300.31
1445.01
Relative Abundance
m/z

Compound 9b

^{1}H-NMR (CDCl₃, 200 MHz)

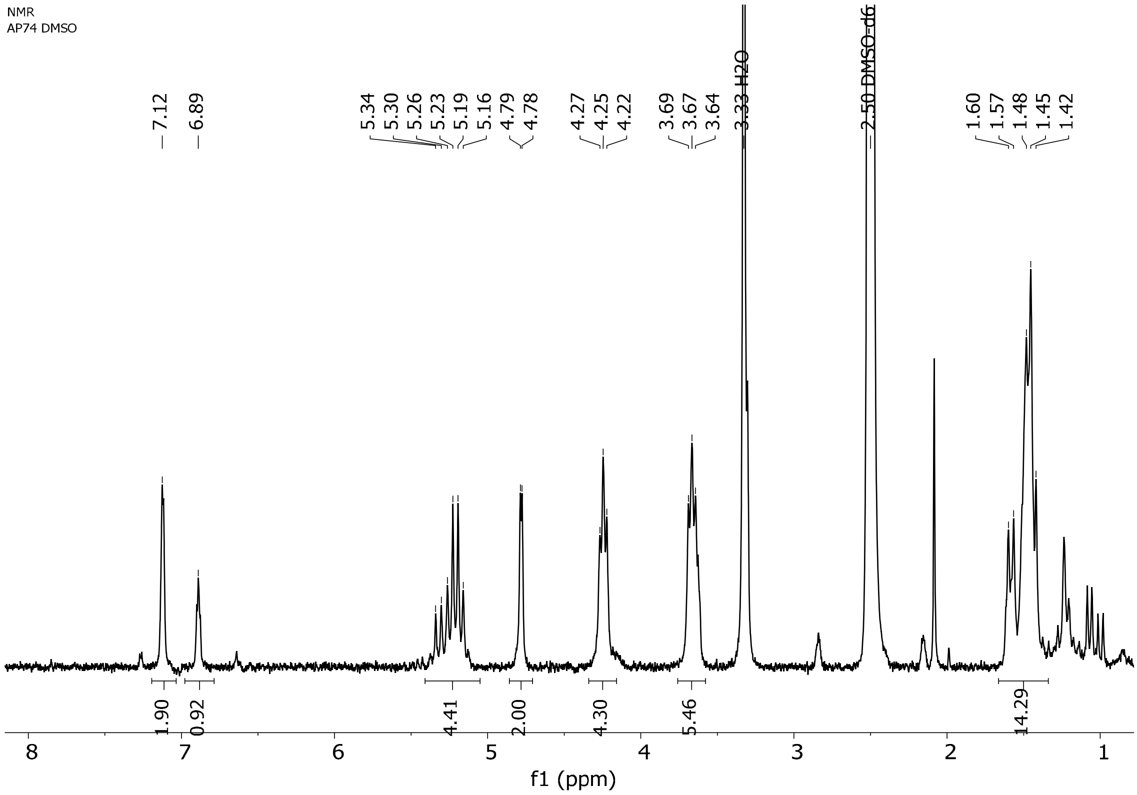

^{13}C-NMR (CDCl₃, 300 MHz)

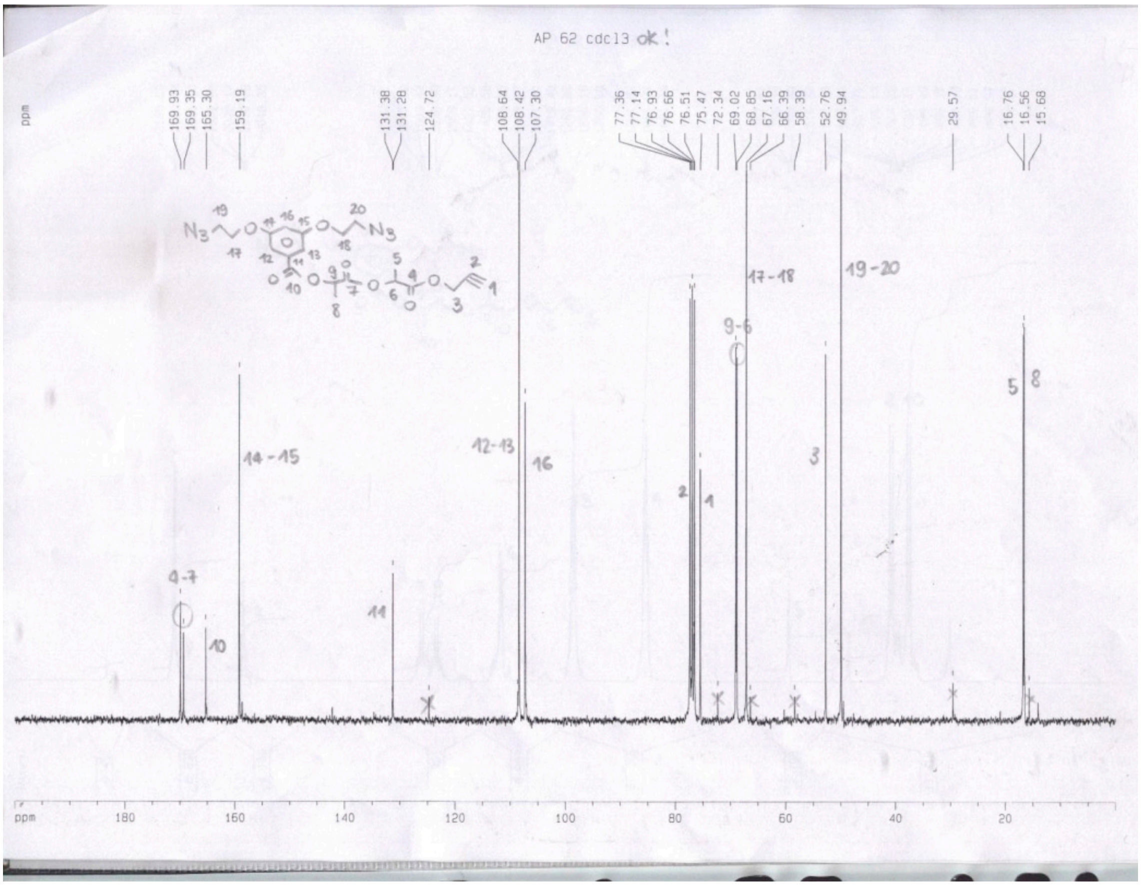

ESI-MS

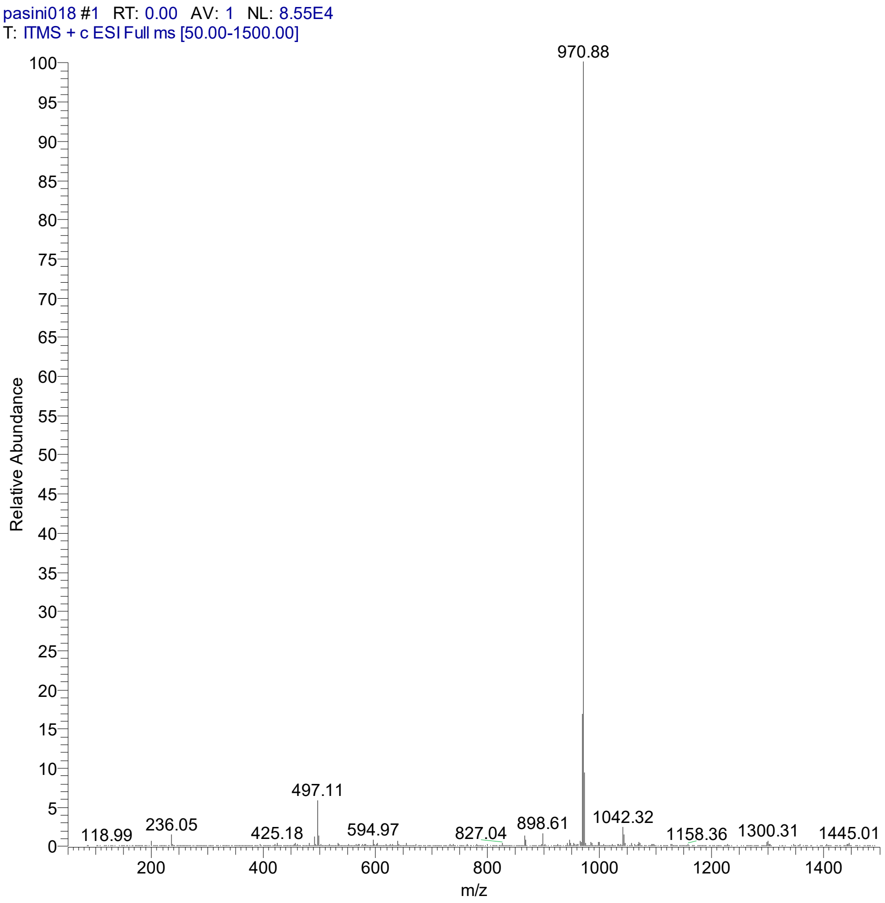

pasini018 #1 RT: 0.00 AV: 1 NL: 8.55E4
T: ITMS + c ESI Full ms [50.00-1500.00]
970.88
497.11
118.99
236.05
425.18
594.97
827.04
898.61
1042.32
1158.36
1300.31
1445.01
Relative Abundance
m/z
